# Efficacy and Safety of Traditional Chinese Medicine for Diabetes: A Double-Blind, Randomised, Controlled Trial

**DOI:** 10.1371/journal.pone.0056703

**Published:** 2013-02-27

**Authors:** Linong Ji, Xiaolin Tong, Hongyuan Wang, Haoming Tian, Huimin Zhou, Lili Zhang, Qifu Li, Yizhong Wang, Hongmei Li, Min Liu, Hongjie Yang, Yanbin Gao, Yan Li, Quanmin Li, Xiaohui Guo, Gangyi Yang, Zhongai Zhang, Zhiguang Zhou, Guang Ning, Yingli Chen, Sanjoy Paul, the Evidence-Based Medical Research of Xiaoke Pill Study Group

**Affiliations:** 1 Peking University People's Hospital, Beijing, China; 2 China Academy of Chinese Medicial Sciences Guang'anmen Hospital, Beijing, China; 3 Peking University School of Public Health, Beijing China; 4 Sichuan University West China Hospital, Chengdu, China; 5 The First Hospital of Hebei Medical University, Shijiazhuang, China; 6 The Third Affiliated Hospital of Peking University of Traditional Chinese and Western Medicine, Beijing, China; 7 The First Affiliated Hospital of Chongqing Medical University, Chongqing, China; 8 The Central Hospital of China Aerospace Corporation, Beijing, China; 9 China Meitan General Hospital, Beijing, China; 10 The First Affiliated Hospital of Guangzhou University of Traditional Chinese Medicine, Guangzhou, China; 11 Shanghai University of Traditional Chinese Medicine Yueyang Hospital of Integrated Traditional Chinese and Western Medicine, Shanghai, China; 12 Beijing University of Traditional Chinese Medicine Dongfang Hospital, Beijing, China; 13 Zhongshan University Sun Yai-sen Memorial Hospital, Zhongshan, China; 14 General Hospital of PLA Second Artillery, Beijing, China; 15 Peking University First Hospital, Beijing, China; 16 The Second Affiliated Hospital of Chongqing Medical University, Chongqing, China; 17 Nanjing Hospital of Traditional Chinese Medicine, Nanjing, China; 18 The Second Xiangya Hospital of Central South University, Changsha, China; 19 Shanghai Jiaotong University Ruijin Hospital, Shanghai, China; 20 Queensland Clinical Trials and Biostatistics Centre, School of Population Health, University of Queensland, Queensland, Australia; Pennington Biomedical Research Center, United States of America

## Abstract

**Background:**

Treatment of diabetes mellitus with Traditional Chinese Medicine has a long history. The aim of this study is to establish the safety and efficacy of traditional Chinese medicine combined with glibenclamide to treat type 2 diabetes mellitus.

**Methods:**

In a controlled, double blind, multicentre non-inferiority trial, 800 patients with unsatisfactory glycemic control (fasting glucose 7–13 mmol/L and HbA1c 7–11%) were randomly assigned to receive Xiaoke Pill, a compound of Chinese herbs combined with glibenclamide, or Glibenclamide in two study groups – drug naive group, and patients previously treated with metformin monotherapy (metformin group). Outcome measures at 48 weeks were the incidence and rate of hypoglycemia, mean difference in HbA1c, and proportion of patients with HbA1c<6.5%.

**Findings:**

In drug naïve group, the total hypoglycemia rate and the mild hypoglycemic episode in the Xiaoke Pill arm were 38% (p = 0.024) and 41% (p = 0.002) less compared to Glibenclamide arm; in Metformin group, the average annual rate of hypoglycemia was 62% lower in Xiaoke Pill arm (p = 0.003). Respective mean changes in HbA1c from baseline were −0.70% and −0.66% for Xiaoke Pill and Glibenclamide, with a between-group difference (95% CI) of −0.04% (−0.20, 0.12) in the drug naïve group, and those in metformin group were −0.45% and −0.59%, 0.14% (−0.12, 0.39) respectively. The respective proportions of patients with a HbA1c level <6.5% were 26.6% and 23.4% in the drug naïve group and 20.1% and 18.9% in the metformin group.

**Interpretation:**

In patients with type 2 diabetes and inadequate glycaemic control, treatment with Xiaoke Pill led to significant reduction in risk of hypoglycemia and similar improvements in glycemic control after 48 weeks compared to Glibenclamide.

**Trial Registration:**

Chinese Clinical Trial Register number, ChiCTR-TRC-08000074

## Introduction

Treatment of diabetes mellitus with Traditional Chinese Medicine (TCM) has a long history of more than 2,000 years in China [1]. Diabetes has been described as “Xiaoke (wasting-thirst)” in Traditional Chinese Medicine [2], [3]. The classic symptoms and signs of “Xiaoke” include sweet urine, dry mouth, thirst, polydipsia, polyorexia, polyphagia, emaciation, and fatigue [4]–[7]. Evidence for glucose-lowing effect of TCM is based mainly on animal studies [8]. Large-scale, direct comparison of TCM and well established allopathic glucose lowering drugs in terms of safety (such as hypoglycemia: one of the most common symptoms during treatment of diabetes) and efficacy has been lacking [9]–[12]. In addition, there is no evidence if TCM has any additional benefits compared with western medicine. One of the major difficulties in studying TCM is the standardizing the dose of TCM in large scale and long-term clinical study.

In China, some diabetes medications are compound preparations of Chinese herbs combined with allopathic medicine such as glibenclamide. One such widely used preparation is Xiaoke Pill [11], which contains 0.25microgram of glibenclamide per pill [12]. The herb components include Radix Puerariae, Radix Rehmanniae, Radix Astragali, Radix Trichosanthis, Stylus Zeae Maydis, Fructus Schisandrae Sphenantherae, and Rhizoma Dioscoreae. These were selected from two ancient TCM formulas for Xiaoke – “Yuquan San” [13] and “Xiaoke Fang” [14]. The selection of those herb components for the treatment of diabetes was based on theory of TCM. Prior studies published in medical journals in China showed significant improvement in diabetes symptoms in favor of Xiaoke Pill. However, the efficacy and safety of Xiaoke Pill has not been evaluated with randomized, double blind, and placebo controlled study.

The Xiaoke Pill is produced with modern pharmaceutical technologies, which ensure the equal quantity of each herb component and glibenclamide in each pill. Therefore, this medicine is suitable for study the efficacy and safety of TCM if controlled properly by glibenclamide.

Evidence-Based Medical Research of Xiaoke Pill is a 52-week, multicentre, double-blind, double-dummy controlled clinical trial to assess the safety and efficacy of Xiaoke Pill compared with glibenclamide alone in patients with inadequate glycemic control and stratified into two groups: newly diagnosed drug naïve patients, and patients treated with metformin for at least three months.

## Methods

### Ethics Statement

The protocol was approved by the Ethics Review Committee affiliated with each study center, and was implemented in accordance with provisions of the Declaration of Helsinki and Good Clinical Practice guidelines. The full names of each ethics committee were listed as follows:

Ethics Committee of The First Hospital of Hebei Medical University, Ethics Committee of The Third Affiliated Hospital of Peking University of Traditional Chinese and Western Medicine, Ethics Committee of Sichuan University West China Hospital, Ethics Committee of The First Affiliated Hospital of Chongqing Medical University, Ethics Committee of The Central Hospital of China Aerospace Corporation, Ethics Committee of Peking University People's Hospital, Ethics Committee of China Meitan General Hospital, Ethics Committee of The First Affiliated Hospital of Guangzhou University of Traditional Chinese Medicine, Ethics Committee of Shanghai University of Traditional Chinese Medicine, Ethics Committee of China Academy of Chinese Medical Sciences Guang'anmen Hospital, Ethics Committee of Beijing University of Traditional Chinese Medicine Dongfang Hospital, Ethics Committee of Zhongshan University Sun Yai-sen Memorial Hospital, Ethics Committee of General Hospital of PLA Second Artillery, Ethics Committee of Peking University First Hospital, Ethics Committee of The Second Affiliated Hospital of Chongqing Medical University, Ethics Committee of Nanjing Hospital of Traditional Chinese Medicine, Ethics Committee of The Second Xiangya Hospital of Central South University, Ethics Committee of Shanghai Jiaotong University Ruijin Hospital.

All the participants provided their written informed consents to participate in this study.

### Study Design

The study consisted of a screening visit, a 4-weeks run-in period, and 48 weeks of follow up (4-weekly) after randomization. Patients deemed eligible at screening entered a 4 weeks run-in period, reinforced by diet and exercise recommendations, to achieve an entry plasma glucose level between 126–234 mg/dl (7–13 mmol/L) and glycated hemoglobin level between 7–11%. At the beginning of the 4-week run-in period, we gave the patients diet and exercise recommendations according to 2007 China Type 2 Diabetes Clinical Practice Guidelines. At the end of the run-in period, according to the protocol, we recorded if the patients control diet and do exercise or not as we recommended. During the run-in period, it will maintain the same normal dose of metformin for patients treated with metformin. Eligible patients were separately randomized to receive double blind and double dummy mono therapy(drug naïve group, n = 400) or add on therapy (metformin group, n = 400) with Xiaoke Pill (Guangzhou Zhongyi Pharmaceutical, Guangzhou, China) or Glibenclamide tablet (Tianjin Pacific Pharmaceutical, Tianjin, China). Of the 18 participant centers in China, 6 were located in TCM hospitals. Statistics Analysis System (SAS) software was used to randomly divide the subjects who have been determined as eligible through screening in the run-in period (Details of Randomization and Blinding were in Supplementary material).

The study management committee consisted of 6 academic members who designed the trial, and one sponsor representative. Data were held by School of Public Health, Peking University, and were independently analyzed by the Queensland Clinical Trials & Biostatistics Centre, University of Queensland.

### Study Population

Through a protocol defined screening process, 1076 patients were initially screed and those were deemed suitable for inclusion in the study. Patients with type 2 diabetes, aged 21–70 years, who met the following inclusion criteria were recruited for the study:1) Drug naïve patients with body mass index (BMI) within 18 kg/m^2^∼28 kg/m^2^; 2) Patients who received treatment with metformin at a stable dose ≥750 mg/day for at least 3 months before screening, with BMI within 18 kg/m^2^∼35 kg/m^2^; 3) Stable body weight within at least 3 months before screening; 4) Poor glycemic control with fasting plasma glucose (FPG) between 126–234 mg/dl (7.0–13 mmol/L) and glycated hemoglobin (HbA1c)>7.0% at screening. Exclusion criteria included FPG≥13 mmol/L or HbA1c≥11%, more than 3 episodes of severe hypoglycemia within 6 months before screening, allergic to sulfonylureas or their ingredients, treatment with glucose-lowing agents other than metformin or insulin within 3 months before screening or with exogenous insulin for more than 1 week within 3 months before screening, a history of heart disease within 1 year before screening, a history of abnormal kidney function or serum creatinine levels reaching the upper limit of normal, alanine aminotransferase (ALT) or aspartate aminotransferase (AST)>2.5 times the upper limit of normal, suffering from acute or chronic hepatitis, hemoglobin disease or chronic anemia, or underlying conditions that could lead to poor compliance.

### Treatment

Metformin doses for patients in the metformin group were maintained by single brand metformin tablet (250 mg/tablet, Beijing Union Pharmaceutical Factory) provided by the study group at enrollment dose levels throughout the study period. The starting dose for Glibenclamide was 1.25 mg/day (half glibenclamide tablet), and for Xiaoke Pill the dose was 5 pills per day. Following the label of Xiaoke Pill, the maximal daily doses were 7.5 mg/day, and 30pills/day, respectively. Study medicines were adjusted every 4 weeks according to FPG levels: 4.4–7.0 mmol/L - no adjustment; >7.0 mmol/L - addition of 5 pills of Xiaoke Pill and/or half tablet of Glibenclamide; <4.4 mmol/L - reduction of 5 pills of Xiaoke Pill and/or half tablet of Glibenclamide. Dosage titration was at the discretion of the investigator, if poorly tolerated.

### Clinical and Biochemical Measurements

Vital signs and any diabetic complication were recorded at every study visit. Anthropometric measurements were taken at baseline and at each of the 13 follow up visits. HbA1c for preliminary screening was measured at participating centers with National Glycohemoglobin Standardization Program (NGSP) certified methods. FPG levels were measured by glucose oxidase method every 4 weeks at participating centers. HbA1c, urinary albumin-creatinine ratio, immunoreactive insulin, C-peptide, high-sensitivity C-reactive Protein (hsCRP), adiponectin and lipids were measured centrally at randomization and 3 monthly intervals. HbA1c was measured by high-performance liquid chromatography (Ultra2 HbA1c Detector, PRIMUS Corporation, USA, normal range: 4 to 6%). An immunonephelometry method was used to measure urinary albumin-creatinine ratio, C-reactive protein, levels of low-density lipoprotein cholesterol (LDL-C), high-density lipoprotein cholesterol (HDL-C), and triglyceride (COBAS Integra 400 Plus System, Roche Diagnostics Ltd. Basel, Switzerland). Immunoreactive insulin and C-peptide were measured by electrochemiluminescence immuno assay (Elecsys 2010 system, Roche Diagnostics Ltd, Basel, Switzerland)). Adiponectin was measured by Enzyme-linked immunosorbent assays (ELISA) [15]. Liver function tests, complete blood count, urine routine assay, kidney function test, twelve-lead ECG, and physical examination were measured or performed at participating centers at recruitment, 24, and 48 weeks after randomization. All study drugs were withheld on the morning of testing.

### Scoring of TCM Symptoms of Diabetes

TCM symptoms of diabetes were evaluated blindly at each visit in 320 study subjects (80 in each treatment arm) who were located at the TCM hospitals only. The symptoms included dry throat and mouth, lack of strength, polyphagia, polydipsia, shortness of breath, vexing heat in the chest, palms and soles, palpitations, insomnia, yellowish urine, and constipation. The questionnaire-based scoring of *TCM symptoms of diabetes* (Table S1) was based on the modification of “Table for quantitative grading of diabetes symptoms” [16]. Each symptom was graded into four categories and assigned a score from 0 to 3 (3 = most severe category).

### Safety and Efficacy Outcomes

The objectives of the trial were to assess the safety and efficacy of Xiaoke Pill compared with glibenclamide alone in patients with inadequate glycemic control and stratified into two groups: newly diagnosed drug naïve patients, and patients treated with metformin for at least three months.

The safety and efficacy outcomes were incidence of hypoglycemia and the change in HbA1c level at 48 weeks respectively. Secondary endpoints included the changes in fasting glucose level, proportion of patients with HbA1c<6.5%, time to reach HbA1c<6.5%, and, β-cell function, insulin resistance levels, fasting lipid profiles, and the TCM symptoms score.

### Safety Assessments

Safety and tolerability were assessed through the analysis of adverse events, laboratory evaluations, and vital signs. Patients were counseled regarding the symptoms of hypoglycemia and requested to immediately perform a finger-stick glucose measurement if any symptoms occurred. Hypoglycemic events were classified as ‘mild’ if the patient had symptoms or a self-measured capillary glucose level (CG)<63 mg/dL (3.5 mmol/L); ‘severe’ if the patient had symptoms with CG<50 mg/dL (2.8 mmol/L), and third party assistance was required; “nocturnal hypoglycemia” if hypoglycemia occurred between falling asleep in the evening and breakfast.

### Statistical Analysis

Xiaoke Pill was regarded as non-inferior to treatment with Glibenclamide only if, after 48 weeks of treatment, the upper limit of the two-sided 95% CI for the difference in mean HbA1c change was less than 0.4%. A sample size of 400 patients was estimated to provide more than 90% power to test the hypothesis that Xiaoke Pill treatment was non-inferior to treatment with Glibenclamide in each of the study groups, assuming a 20% early discontinuation rate, and an expected inter-patient SD of 1.2%.

The primary analysis was conducted in the intention-to-treat (ITT) population, with supportive per-protocol (PP) analysis [17]. Twenty five imputations for missing clinical and biochemical measurements were performed using multiple-imputation technique based on Bayesian MCMC method [18]. The highest proportion of missing data on longitudinal measurements was only 4%. To account for center-level clustering, the study centers were included as random effect in all regression models. For normal continuous variables, mixed linear regression models were used [19]. Mixed-effect logistic regression models were used to analyze categorical outcomes, with adjustment for baseline data. The ‘unstructured’ covariance structure was used for all mixed-effect models. The primary efficacy end point was change from baseline HbA1c at week 48; end point analysis at week 24 was exploratory. Difference in the estimated marginal means between the treatment arms along with bootstrapped 95% CI was estimated to draw inference on non-inferiority. The proportion of patients with hypoglycemia was analyzed using a generalized binomial model without adjustment for covariates. For the comparison of hypoglycemia rates, generalized regression models with zero-inflated negative binomial distribution were used.

The insulin sensitivity and insulin resistance measures, and the study doses were analyzed with the use of generalized mixed-effect models with gamma distribution. The symptom score data were analyzed using non-parametric quintile regression, with treatment comparisons at median levels of the symptom scores. All regression fits were bootstrapped for 1000 times to obtain the bootstrapped standard errors. Time to reach HbA1c<6.5% was summarized using Kaplan–Meier estimates. SAS 9.2 (SAS Inc.) and STATA 11 software were used for all statistical analyses.

The protocol for this trial and CONSORT checklist are available as supporting information (Protocol S1 and Checklist S1). Trial Registration: Chinese Clinical Trial Register number, ChiCTR-TRC-08000074.

## Results

### Study Characteristics

**Figure 1:**
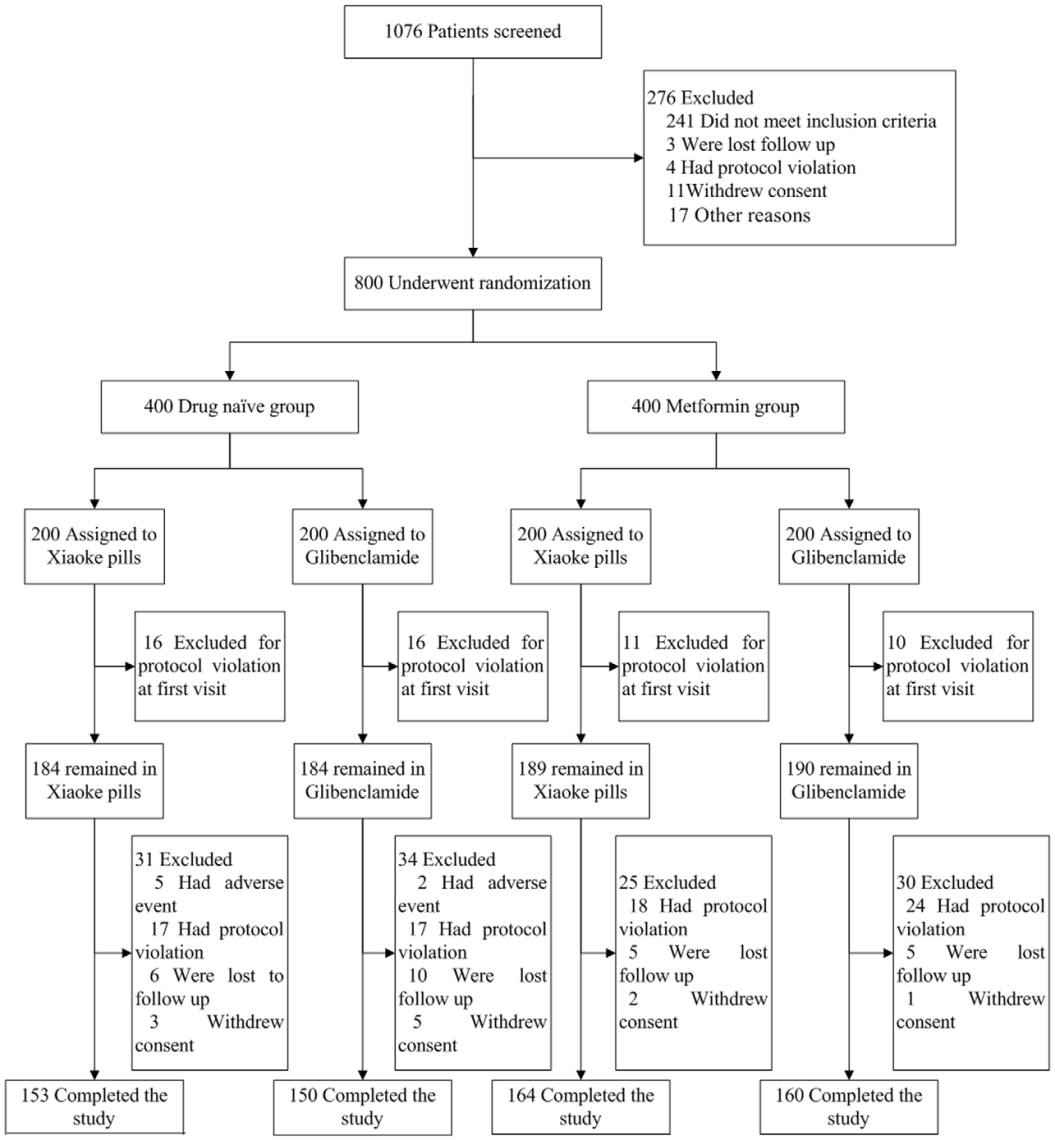
Enrollment and Outcomes.

The patients were enrolled between Dec 20^th^, 2007 and Oct 17^th^, 2008. 1076 patients who met the inclusion criteria were recruited for the study. After the end of run-in, 800 patients who still meet the inclusion criteria were randomly divided into the drug naïve and metformin study groups (Fig. 1). The respective mean ±SD ages were 53.7±8.6 and 54.7±8.8 years. The median (IQR) duration of diabetes in metformin group was 3 (1.0, 6.3) years; based on Mann-Whitney U tests, no significant differences were observed in the baseline variables between the two treatments in two study groups (Table 1). The total numbers of patients who did not complete the study did not differ significantly – 16.8% and 18.5% in Xiaoke Pill and Glibenclamide arms, respectively, within drug naïve group (n = 65); and 13.6% and 16.3%, respectively, within metformin group (n = 55). Demographic, anthropometric, and metabolic characteristics of these lost-to-follow-up patients did not differ from those who completed the study.

**Table 1:** Characteristic of the Patients at Baseline.

Variable	Drug Naïve Group			Metformin Group		
	Xiaoke Pill	Glibenclamide	p	Xiaoke Pill	Glibenclamide	p
	(n = 184)	(n = 184)		(n = 189)	(n = 190)	
**Demographics Characteristics**						
Age — yr	54±9	54±8	0.91	54±8	55±9	0.94
Male — no. (%)	104(56.5)	106(57.6)	0.83	100(52.9)	92(48.4)	0.38
Patient in CTH— no. (%)	76(41.3)	74(40.2)	0.87	74(39.2)	76(40.0)	0.85
Excercise— no. (%)	135(73.4)	147(79.9)	0.14	146(77.2)	156(82.1)	0.24
Diet— no. (%)	138(75.0)	155(84.2)	0.028	157(83.1)	166(87.4)	0.24
**Anthropometric Characteristics**						
Weight (kg)	67.1±9.5	66.8±10.5	0.51	68.7±10.4	67.1±11.0	0.48
Body-mass index (kg/m^2^)	24.5±2.2	24.4±2.5	0.69	25.4±2.9	25.0±3.1	0.63
Waist-Hip Ratio	0.9±0.1	0.9±0.1	0.89	0.9±0.1	0.9±0.1	0.90
**Blood Pressure** — mm Hg						
Systolic	126±12	125±14	0.36	125±12	126±13	0.49
Diastolic	78±8	78±9	0.59	78±8	77±8	0.54
Anti-Hypertensive— no. (%)	33(17.9)	28(15.2)	0.48	38(20.1)	31(16.3)	0.34
**Metabolic Characteristics**						
FPG—mmol/L	9.16±1.74	8.98±1.59	0.31	8.91±1.56	9.09±1.84	0.29
Glycated hemoglobin—%	7.92±1.47	7.85±1.54	0.57	7.68±1.39	7.90±1.51	0.18
Fasting insulin—mIU/L^a^	8.10 (4.98–11.84)	7.96 (5.31–11.69)	0.87	8.40 (5.31–12.13)	7.60 (5.30–11.92)	0.46
HOMA- IS	38.40±31.11	39.76±33.85	0.53	37.96±26.82	38.01±32.36	0.41
HOMA-IR	4.13±4.19	4.19±3.73	0.11	3.75±2.58	3.92±3.50	0.17
**Lipids**						
Total cholesterol —mmol/L	4.99±0.98	4.97±1.17	0.87	5.14±2.08	4.96±0.94	0.26
High-density lipoprotein— mmol/L	1.1±0.3	1.1±0.3	0.49	1.1±0.3	1.2±0.3	0.53
Low-density lipoprotein— mmol/L	3.20±0.85	3.06±0.92	0.12	2.96±0.87	3.03±0.87	0.23
Triglycerides— mmol/L^a^	1.65 (1.38–2.31)	1.79 (1.25–2.63)	0.50	1.87 (1.41–2.92)	1.87 (1.47–2.54)	0.46
Ratio of albumin to creatinine^a^	3.53 (0.96–10.40)	3.16 (0.86–11.19)	0.11	3.88 (0.93–11.70)	3.94 (0.86–15.94)	0.28
ALT—U/L^a^	20.4 (15, 30)	22 (15.1, 33)	0.44	23 (16, 34)	23 (16, 31)	0.23
AST—U/L^a^	21 (17, 27.5)	21 (17, 27.6)	0.78	21 (17, 28)	21 (17, 27.7)	0.79
*Score of TCM symptoms of diabetes*	8 (6,9)	8.5 (6,10)	0.27	8.5 (7,12)	8 (6,11)	0.22

a Median (Inter Quartile Range).

**Figure 2:**
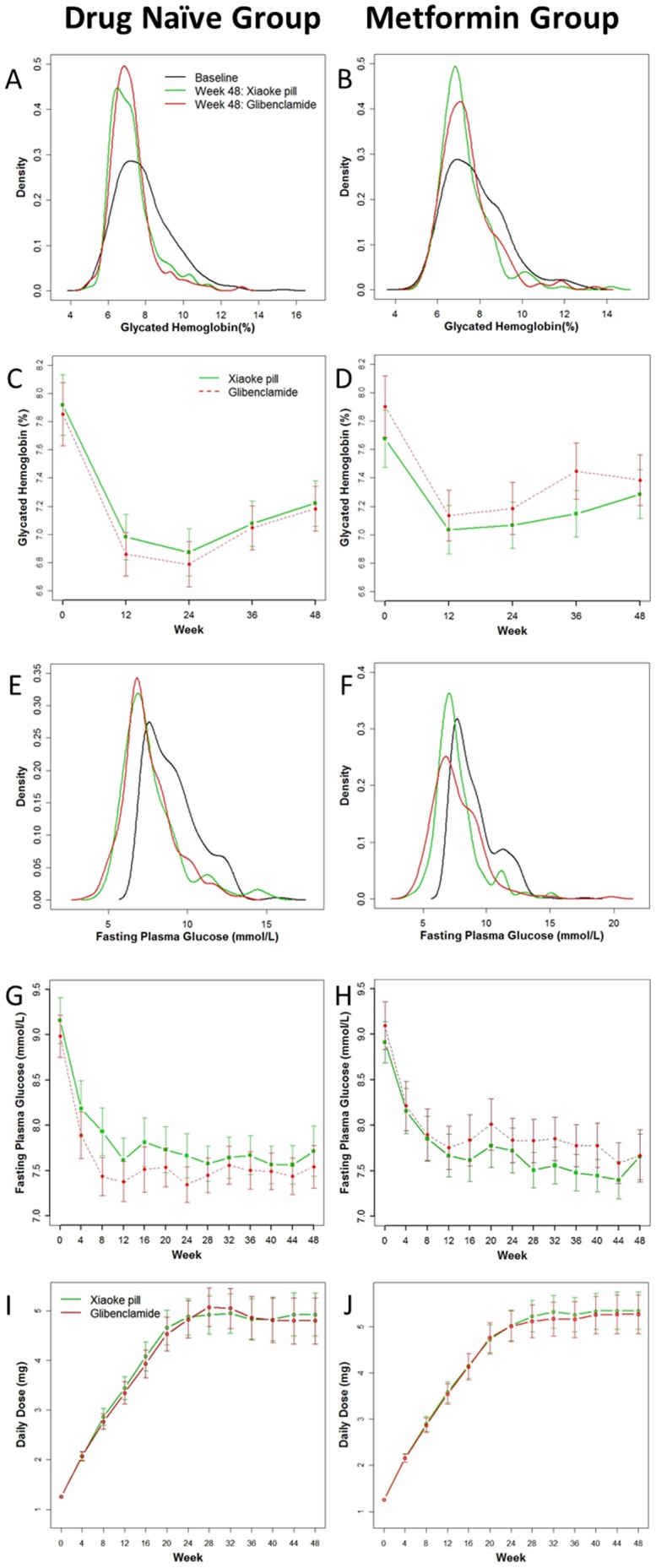
Primary and secondary outcomes over 48 weeks.

The glibenclamide doses per day were similar in the two treatment arms during 48 weeks of follow-up (Fig. 2 I&J). The mean ± SD dose of metformin was 1140±340 mg and 1130±360 mg, in Xiaoke Pill and Glibenclamide arms, respectively. The baseline distributions of HbA1c and FPG were similar in both study groups (Fig. 2 A& B, 2 E & F).

### Outcome in Drug Naïve Group

All safety analyses were done on the ITT population. Patients in the Xiaoke Pill arm were 38% less likely to have any hypoglycemia compared to those in the Glibenclamide arm [OR (95% CI): 0.62 (0.42, 0.94), p = 0.024]. The average annual rate of hypoglycemia was 24% lower in patients treated with Xiaoke Pill [Rate Ratio (95% CI): 0.76 (0.49, 1.18)]. Patients in Xiaoke Pill arm were also 41% less likely to have a mild hypoglycemic episode compared to those in the Glibenclamide arm [OR (95% CI): 0.59 (0.42, 0.82), p = 0.002, Table 2].

**Table 2:** Outcomes and changes from baseline.

	Treatment Naïve Group			Metformin Group		
	Xiaoke Pill	Glibenclamide	Difference in Mean Change (95% CI)	Xiaoke Pill	Glibenclamide	Difference in Mean Change (95% CI)
HbA1c— %						
Mean at 24 week (95% CI)	6.84 (6.68, 7.01)	6.77 (6.51, 7.03)		7.05 (6.86, 7.25)	7.17 (6.93, 7.41)	
Absolute change from baseline at 24 week	−1.07 (−1.39, −0.76)	−1.08 (−1.41, −0.74)	0.01 (−0.12, 0.13)	−0.65 (−0.86, −0.45)	−0.78 (−1.12, −0.43)	0.12 (−0.13, 0.38)
Mean at 48 week (95% CI)-ITT	7.20 (7.01, 7.39)	7.18 (6.91, 7.45)		7.27 (7.04, 7.50)	7.36 (7.15, 7.57)	
Absolute change from baseline-ITT	−0.70 (−1.06, −0.34)	−0.66 (−1.05, −0.28)	−0.04 (−0.20, 0.12)	−0.45 (−0.68, −0.22)	−0.59 (−0.94, −0.23)	0.14 (−0.12, 0.39)
Mean at 48 week (95% CI)-PP	6.79 (6.62, 6.96)	6.85 (6.65, 7.04)		6.94 (6.73, 7.15)	6.97 (6.69, 7.24)	
Absolute change from baseline-PP	−0.87 (−1.37, −0.37)	−0.71 (−1.09, −0.33)	−0.16 (−0.48, 0.16)	−0.41 (−0.58, −0.25)	−0.57 (−0.93, −0.21)	0.16 (−0.18, 0.49)
Total hypoglycemia— no. (%)	28(15.2)	39(21.2)		23(12.2)	29(15.3)	
Odd Ratio	0.62(0.42,0.94)		p = 0.024	0.76(0.43,1.35)		p = 0.35
Average Rate/Patient/Year	2.61(1.73)	3.00(1.97)		1.91(1.24)	3.76(2.95)	
Rate Ratio	0.76 (0.48, 1.18)		p = 0.22	0.38 (0.20, 0.71)		p = 0.003
Mild hypoglycemia— no. (%)	26(14.1)	38(20.7)		23(12.2)	28(14.7)	
Odd Ratio	0.59(0.42, 0.82)		p = 0.002	0.80(0.44, 1.44)		p = 0.45
Average Rate/Patient/Year	2.58 (1.50)	2.95 (1.90)		1.87 (1.25)	3.54 (4.01)	
Rate Ratio	0.76 (0.49, 1.18)		p = 0.22	0.40 (0.20, 0.78)		p = 0.007
Nocternal hypoglycemia— no. (%)	3(1.6)	4(2.2)		1(0.5)	8(4.2)	
Odd Ratio	0.75(0.16, 3.33)		p = 0.93	0.12(0.01, 0.98)		p = 0.037
Average Rate/Patient/Year	2 (1)	1.25 (0.5)		1	1.25 (0.71)	
Rate Ratio	-	-		-	-	
N(%)of patients reaching A1c <6.5% at 48 week	49 (26.6)	43 (23.4)		38 (20.1)	36 (18.9)	
Odds Ratio (95% CI)	1.31 (0.81, 2.13)		p = 0.37	1.20 (0.75, 1.92)		p = 0.44
FPG—mmol/L						
Mean at 48 week (95% CI)	7.72 (7.32, 8.11)	7.54 (7.22, 7.86)		7.71 (7.41, 8.01)	7.73 (7.41, 8.04)	
Absolute change from baseline	−1.42 (−1.79, −1.06)	−1.43 (−1.79, −1.06)	0.01 (−0.39, 0.40)	−1.31 (−1.71, −0.90)	−1.48 (−1.87, −1.08)	0.17 (−0.18, 0.51)
N(%)of patients reaching FPG<7 mmol/L	78 (42.4)	82 (44.6)		69 (36.5)	79 (41.6)	
Odds Ratio (95% CI)	1.05 (0.70, 1.58)		p = 0.81	0.72 (0.48,1.08)		p = 0.11
Weight(kg)						
Mean at 48 week (95% CI)	68.09 (65.76, 70.41)	67.75 (65.41, 70.10)		68.99 (66.68, 71.29)	67.91 (64.55, 71.28)	
Absolute change from baseline	0.99 (0.19, 1.79)	1.14 (0.90, 1.38)	−0.15 (−0.89, 0.59)	0.51 (0.08, 0.93)	0.83 (0.48, 1.18)	−0.32 (−0.71, 0.06)
Systolic blood pressure — mm Hg						
Mean at 48 week (95% CI)	126 (124, 128)	126 (124, 129)		125 (123, 127)	128 (124, 131)	
Absolute change from baseline	0.33 (−1.64, 2.29)	1.63 (−0.41, 3.69)	−1.31 (−2.97,0.35)	−0.11 (−2.54, 2.32)	0.82 (−2.07, 3.71)	−0.93 (−3.64, 1.78)
Diastolic blood pressure — mm Hg						
Mean at 48 week (95% CI)	77(75, 78)	77 (76, 79)		78 (76, 79)	78 (76, 79)	
Absolute change from baseline	−0.88 (−2.17, 0.40)	−0.54 (−1.98, 0.91)	−0.35 (−2.33, 1.63)	−0.98 (−2.40, 0.44)	−0.04 (−1.64, 1.56)	−0.94 (−2.98, 1.10)
Total cholesterol —mmol/L						
Mean at 48 week (95% CI)	4.95 (4.80, 5.11)	4.67 (4.51, 4.83)		5.21 (5.02, 5.39)	4.84 (4.64, 5.03)	
Absolute change from baseline	−0.02 (−0.15, 0.11)	−0.30 (−0.54, −0.07)	0.28 (0.02, 0.54)	0.07 (−0.21, 0.34)	−0.13 (−0.28, 0.02)	0.20 (−0.10, 0.49)
Low-density lipoprotein— mmol/L						
Mean at 48 week (95% CI)	2.96 (2.78, 3.15)	2.69 (2.54, 2.84)		2.88 (2.69, 3.08)	2.79 (2.62, 2.97)	
Absolute change from baseline	−0.22 (−0.38, −0.07)	−0.38 (−0.51, −0.23)	0.15 (−0.03, 0.32)	−0.08 (−0.22, 0.06)	−0.31 (−0.46, −0.16)	0.23 (0.06, 0.40)
High-density lipoprotein— mmol/L						
Mean at 48 week (95% CI)	1.06 (1.00, 1.12)	1.05 (1.00, 1.10)		1.07 (1.01, 1.13)	1.12 (1.07, 1.16)	
Absolute change from baseline	−0.08 (−0.11, −0.04)	−0.07 (−0.11, −0.02)	−0.01 (−0.06, 0.03)	−0.02 (−0.07, 0.03)	−0.05 (−0.10, 0.001)	0.03 (−0.03, 0.09)
Triglycerides— mmol/L						
Mean at 48 week (95% CI)	1.97 (1.79, 2.14)	2.03 (1.83, 2.23)		2.43 (2.09, 2.77)	1.87 (1.70, 2.05)	
Absolute change from baseline	−0.12 (−0.30, 0.06)	−0.32 (−0.58, −0.06)	0.20 (−0.09, 0.49)	−0.15 (−0.51, 0.22)	−0.39 (−0.57, −0.20)	0.24 (−0.24, 0.72)
C-peptide— ug/L						
Mean at 48 week (95% CI)	2.22 (1.90, 2.55)	2.34 (2.06, 2.61)		2.29 (2.03, 2.56)	2.08 (1.85, 2.30)	
Absolute change from baseline	−0.12 (−0.37, 0.13)	0.02 (−0.17, 0.21)	−0.14 (−0.41, 0.12)	−0.02 (−0.20, 0.16)	−0.10 (−0.24, 0.05)	0.08 (−0.08, 0.23)
Fasting insulin—mIU/L						
Mean at 48 week (95% CI)	9.50 (8.13, 10.88)	10.68 (9.53, 11.83)		10.07 (9.26, 10.89)	9.74 (8.79, 10.69)	
Absolute change from baseline	−0.53 (−1.53, 0.48)	0.33 (−1.43, 2.10)	−0.86 (−2.79, 1.07)	0.47 (−0.81, 1.75)	−0.10 (−0.81, 0.62)	0.56 (−0.79, 1.92)
HOMA-IR						
Mean at 48 week (95% CI)	3.26 (2.72, 3.80)	3.62 (3.17, 4.06)		3.51 (3.10, 3.91)	3.27 (2.96, 3.58)	
Absolute change from baseline	−0.84 (−1.34, −0.33)	−0.56 (−1.26, 0.14)	0.28 (−0.55, 1.11)	−0.30 (−0.95, 0.34)	−0.72 (−1.09, −0.35)	0.41 (−0.28, 1.10)
HOMA- B						
Mean at 48 week (95% CI)	48.87 (32.94, 64.80)	64.95 (54.69, 75.21)		55.08 (50.66, 59.51)	60.15 (49.18, 71.13)	
Absolute change from baseline	20.50 (12.21, 28.80)	24.77 (14.40, 35.14)	−4.27 (−18.28, 9.74)	16.75 (12.51, 20.99)	21.43 (12.01, 30.85)	−4.68 (−14.75, 5.39)
Score of TCM symptoms of diabetes						
Median (IQR) at 48 week	2 (1,4)	4 (3,5)		3 (2,4)	3.5 (2,5)	
Absolute change from baseline	−5.64 (−6.4, −4.9)	−4.68 (−5.46, −3.89)	−0.96 (−2.08, 0.15)	−6.38 (−7.10, −5.65)	−4.73 (−5.4, −4.03)	−1.65 (−2.66, −0.64)

The maximal reduction in the mean HbA1c level occurred by 24 weeks and then increased marginally for both treatments (Fig. 2 C). The mean change from baseline in HbA1c at week 48 was −0.70% in the Xiaoke Pill group and −0.66% in the Glibenclamide group (Table 2), with a between-group difference (95% CI) of −0.04% (−0.20, 0.12). The upper-limit of the 95% CI for the between-group mean difference met the pre-specified criterion for declaring non-inferiority of Xiaoke Pill to Glibenclamide. In PP subjects, the between-group difference (95% CI) was −0.16 (−0.48, 0.16).

At 48 week, patients were 31% more likely to reduce HbA1c below 6.5% in the Xiaoke Pill group [OR (95% CI): 1.31(0.81, 2.13)]. The proportion of patients with HbA1c<7% was the same in the two treatment arms [89 (48.4%)].The mean (95% CI) weeks to reach HbA1c<6.5% was 29.1 (26.4, 31.8) and 28.0 (25.3, 30.8) weeks, in Xiaoke Pill and Glibenclamide arms respectively. At 48 week, the significant reduction in the FPG level was similar in both treatment arms (Fig. 2E and Table 2).

A significant reduction of total cholesterol by 0.28 mmol/L was observed in the Glibenclamide group (CI of between-group difference: 0.02–0.54 mmol/L). The symptom, as evaluated by the score of *TCM symptoms of diabetes*, was lower in the Xiaoke Pill arm with between-group difference of −0.96 (95% CI: −2.08, 0.15).

### Outcome in Metformin Group

Patients in Xiaoke Pill arm were 24% less likely to have any hypoglycemia compared to those in the Glibenclamide arm [OR (95% CI): 0.76 (0.43, 1.35)]. The average annual rate of hypoglycemia was 62% lower in patients treated with Xiaoke Pill [Rate Ratio (95% CI): 0.38 (0.20, 0.71), p = 0.003]. There was no significant difference in the incidence of mild hypoglycaemia between the treatment arms. Eight patients reported nocturnal hypoglycemia in the Glibenclamide arm, compared to only one in the Xiaoke Pill arm.

Maximal reduction in the average HbA1c level occurred by 12 week (Fig. 2 C). The mean reduction from baseline in HbA1c at week 48 was 0.45% in the Xiaoke Pill arm and 0.59% in the Glibenclamide arm, with a between group difference (95% CI) of 0.14 (−0.12, 0.39)% (Table 2). The upper-limit of the 95% CI for the between-group mean difference in change from baseline in HbA1c marginally met the pre-specified criterion for declaring non-inferiority of Xiaoke Pill.

At 48 week, 20.1% and 18.9% patients had HbA1c below 6.5% in the Xiaoke Pill and Glibenclamide arm, respectively. The proportion of patients with HbA1c<7% at 48 weeks was higher in the Xiaoke Pill arm [89(47.1%)] compared to those in the Glibenclamide arm [77 (40.5%)], and mean (95% CI) weeks to reach HbA1c<6.5% was 31.1 (28.3, 33.9) and 30.6 (27.8, 33.4) weeks, respectively. The significant reduction in the FPG level was similar in both treatment arms (Fig. 2F and Table 2).

Significant reduction in LDL-C and triglyceride levels, by 0.31 and 0.39 mmol/L, respectively, were observed in the Glibenclamide arm. However, the CI of between-group difference in triglyceride was −0.24, 0.72 mmol/L. The TCM diabetes symptom score was significantly lower in the Xiaoke Pill arm with between-group difference of −1.65 (95% CI: −2.66, −0.64).

### Adverse Events

No serious adverse event was reported during the study. The proportions of adverse events, which were reported in only four categories, did not differ significantly (Table S2). For those with dyslipidemia, the average levels of cholesterol and triglyceride did not differ significantly between the treatments.

## Discussion

Our study showed that Xiaoke Pill is associated with reduced risk of hypoglycemia and has similar glucose-lowing efficacy as compared with glibenclamide. At the same level of glibenclamide dose and glycemic control, the Xiaoke Pill had clearly demonstrated a reduced incidence and rate of hypoglycemia as compared with glibenclamide, suggesting that the TCM in the Xiaoke Pill had protective effects against hypoglycemia induced by glibenclamide. The herb components in Xiaoke pill include Radix Puerariae, Radix Rehmanniae, Radix Astragali, Radix Trichosanthis, Stylus Zeae Maydis, Fructus Schisandrae Sphenantherae, an d Rhizoma Dioscoreae. A recent study showed Radix astragali, which is one of the TCM components of Xiaoke Pill, could amplify the glucose counter-regulatory response to insulin-induced hypoglycemia in rats [20]. Radix astragali might exert its action directly, or indirectly, in two brain regions, the paraventricular hypothalamus (PVN) and nucleus tractus solitaries (NTS), known to be involved in glucose-sensing during hypoglycemia [20]. One Chinese study found Radix Puerariae could protect neuron from hypoglycemic and hypoxia damage in vitro cell experiment [21]. However, most of the studies focus on the anti-oxidative effect of the traditional Chinese herbs. More studies are needed to interpret the mechanism of TCM's anti-hypoglycemia effect.

Between 4 and 24 weeks of the study, 97 (52.7%) and 95 (51.6%) patients in the treatment naïve group reduced HbA1c below 6.5% in the Xiaoke Pill and Glibenclamide arms, respectively. During same period, 129 (70.1%) and 125 (67.9%) patients reduced FPG below 7 mmol/L. At 48 week, the absolute changes in FPG levels were also similar in the two treatment arms in both study groups (Fig. S1). Our study design allowed comparing the TCM in the Xiaoke Pill to its placebo on top of equal amount of glibenclamide. Given the label limitation on maximum dosage of Xiaoke Pill, the low dose usage of glibenclamide in this trial gave more opportunity of detecting the glucose-lowing effect of TCM.

Five prior studies published in medical journals in China [22]–[26] showed significant improvement in diabetes symptoms in favor of Xiaoke Pill. These 4–8 weeks open-level studies were mostly single centre studies, and only one study assessed the hypoglycemic events. However, the validity of the design of these studies is questionable, and makes it hard to draw any robust conclusion with regard to safety and efficacy of Xiaoke Pill [27].

Xiaoke Pill was associated with greater improvement in symptoms of diabetes in a pre-specified subgroup analysis. However, caution should be taken in interpreting this result, as improvement in symptoms might also be associated with the reduction of hypoglycemia risk observed in Xiaoke Pill treatment arm. Symptoms associated with hypoglycemia such as fatigue, hunger, and palpitation were included in the score system. Further study is needed to validate this finding with the consideration that this finding might be specific to clinical studies involving glucose lowing agents that could cause hypoglycemia.

No differences were observed in changes in insulin resistance, β-cell function, HDL, triglyceride, blood pressure, hsCRP and adiponectin (data not presented) between Xiaoke Pill and Glibenclamide treatment arms. Such results suggest that TCM in Xiaoke pill had no additional impact on the pathophysiological changes associated with type 2 diabetes.

TCM is used widely in treating type 2 diabetes not only in China, but also in the other parts of the world. But the role of TCM and other herbal medicines in the management of type 2 diabetes is still not established [28]. The absence of scientific understanding has caused skepticism and criticism about TCM, often because of the low methodological quality of trials [8], [9], [11], [29], [30]. Furthermore, most of these trials were published in Chinese and were not included in systematic reviews. Selective publication of positive trials is another problem. Over 80% [28] of people in developing countries depend on herbal medicine for basic health care. Thus the evidence for safety and efficacy of TCM use for treatment of diabetes, provided by this study, is a contribution towards reducing entrenched inequity in access to effective care for poor people.

## Supporting Information

Table S1 Score of TCM symptoms of diabetes.
(DOC)

Table S2 Adverse Events.
(DOC)

Figure S1 **Mean (95% CI) percentage change at 48 weeks from baseline in Glycated Hemoglobin, Fasting Plasma Glucose and Symptom Score.**
(DOC)

Checklist S1 **CONSORT checklist.**
(DOC)

Protocol S1 **Trial protocol.**
(DOC)

Appendix S1 **List of Investigators.**
(DOC)
