# Supplementary material for: Efficacy and Safety of Traditional Chinese Medicine for Diabetes: A Double-Blind, Randomised, Controlled Trial
Source: PLoS One. 2013 Feb 27;8(2):e56703. doi: 10.1371/journal.pone.0056703 (PMC3584095; doi:10.1371/journal.pone.0056703)
Supplement: Table S1 — Score of TCM symptoms of diabetes. (DOC) [file pone.0056703.s001.doc]

**Table S1: Score of TCM symptoms of diabetes**

| **Symptom** | **Mild（1point）** | **Moderate（2 point）** | **Severe（3 point）** |
| --- | --- | --- | --- |
| **Dry mouth and throat** | Occasionally | Some times | Often |
| **Fatigue** | Able to do daily work | Hard to do daily work | Unable to do daily work |
| **Polyphagia and easily hungry** | Only happen before meal | Happen at any time | Happen at any time accompanied by hypoglycemia symptoms |
| **Thirsty for drink** | Increased water intake <500ml | 500ml<Increased water intake <1000ml | Increased water intake >1000ml |
| **Short of breath, lazy to talk** | Happen after heavy work | Happen after daily work | Happen at any time |
| **Vexation** | Occasionally | Some times | Often |
| **Feverish palms and soles** | Occasionally | Some times | Often |
| **Palpitation** | Occasionally | Some times | Often |
| **Insomnia** | 4h/day < Sleeping time < 6h/day | 2h/day < Sleeping time <4h/day | Sleeping time <2h/day |
| **Constipation** | Dry stool, defecate everyday | Dry stool, defecate every 2-3days | Dry stool, defecate >every 3 days |

Note: Score as “0” if there are no symptoms
