# Supplementary material for: Efficacy and Safety of Traditional Chinese Medicine for Diabetes: A Double-Blind, Randomised, Controlled Trial
Source: PLoS One. 2013 Feb 27;8(2):e56703. doi: 10.1371/journal.pone.0056703 (PMC3584095; doi:10.1371/journal.pone.0056703)
Supplement: Table S2 — Adverse Events. (DOC) [file pone.0056703.s002.doc]

**Table S2: Adverse Events**

Listed adverse events are those that occurred in any patient in any of the study groups.

| Event | Treatment Naïve Group | |  | Metformin Group | |  |
| --- | --- | --- | --- | --- | --- | --- |
|  | Xiaoke Pill | Glibenclamide | p | Xiaoke Pill | Glibenclamide | p |
| Urinary tract Infection | 5 (2.7) | 4 (2.2) | 0.75 | 0 | 3 (1.6) | - |
| Upper Respiratory Tract Infection | 9 (4.9) | 10 (5.4) | 0.81 | 15 (7.9) | 8 (4.2) | 0.14 |
| Elevated ALT/AST | - | 1 (0.5) | - | 6 (3.2) | 3 (1.6) | 0.34 |
| Dyslipidemia | 18 (9.8) | 18 (9.8) | 1 | 21 (11.1) | 11(5.8) | 0.06 |
