# Supplementary material for: Efficacy and Safety of Traditional Chinese Medicine for Diabetes: A Double-Blind, Randomised, Controlled Trial
Source: PLoS One. 2013 Feb 27;8(2):e56703. doi: 10.1371/journal.pone.0056703 (PMC3584095; doi:10.1371/journal.pone.0056703)
Supplement: Figure S1 — Mean (95% CI) percentage change at 48 weeks from baseline in Glycated Hemoglobin, Fasting Plasma Glucose and Symptom Score. (DOC) [file pone.0056703.s003.doc]

| Drug Naïve Group | Metformin Group |
| --- | --- |
|  |  |

**Figure S1: Mean (95% CI) percentage change at 48 weeks from baseline in Glycated Hemoglobin, Fasting Plasma Glucose and Symptom Score.**
