# Supplementary material for: Efficacy and Safety of Traditional Chinese Medicine for Diabetes: A Double-Blind, Randomised, Controlled Trial
Source: PLoS One. 2013 Feb 27;8(2):e56703. doi: 10.1371/journal.pone.0056703 (PMC3584095; doi:10.1371/journal.pone.0056703)
Supplement: Protocol S1 — Trial protocol. (DOC) [file pone.0056703.s005.doc]

**Evidence-Based Medical Research of Zhongyi Xiaoke Pills**

——A Multicenter, randomized, double blind and double dummy, masculine parallel group trial

**Clinical Trial Protocol**

Trial title: Evidence-Based Medical Research of Zhongyi Xiaoke Pills

Investigational drug generic name: Xiaoke Pills

Protocol discussion date: September 2007

Protocol finalization date: October 2007

Unit in charge of the trial: Peking University People’s Hospital (Seal)

Principal: Ji Linong (Signed)

Data management and statistical analysis: Peking University School of Public Health

Statistics manager: Wang Hongyuan Tel: 010-82801619

Clinical trial audit unit: Guangzhou Boji Clinical Research Center of New Drugs

Project contact: Wang Shuaishuai Tel: 020-38473208

Center testing audit unit: Beijing VPS-CRO Group

Project contact: Dai Chengxiang Tel: 010-88500088-284

Sponsor: Guangzhou Zhongyi Pharmaceutical Co., Ltd. (Seal)

Address: 11th Floor, West Tower, Times Square, No. 28 Tianhe North Road, Guangzhou

Person in charge: Wu Changhai (Signed)

Contact: Deng Huimin, Yang Longfei Tel: 020-82091963

Chinese Diabetes Society of Chinese Medical Association recommends using the WHO (1999) diagnostic criteria in population of China.

| 1. Diabetes symptoms + plasma glucose level at any time ≥11.1 mmol/l (200 mg/dl) or  2. Fasting plasma glucose (FPG) level ≥ 7.0 mmol/l (126 mg/dl)  3. OGTT test showing 2-hour PG level ≥ 11.1mmol/l (200 mg/dl) |
| --- |

Explanation:

In the above table, plasma glucose level refers to venous plasma glucose level, which is determined by glucose oxidase method. It is recommended to determine the venous plasma glucose values. If it uses the finger whole blood and (or) venous whole blood to measure the glucose values, there will be some changes in the division point of diagnosis. See the table below:

Diagnostic criteria for blood glucose of diabetes and IGT/IFG

|  | Blood glucose concentration [mmol/L(mg/dl)] | | |
| --- | --- | --- | --- |
|  | Whole blood | | Venous plasma |
|  | Venous | Capillary (finger) |
| Diabetes |  |  |  |
| Fasting | ≥6.1(110) | ≥6.1(110) | ≥7.0(126) |
| Or 2 hours after the load Or both | ≥10.0(180) | ≥11.1(200) | ≥11.1(200) |
| Impaired glucose tolerance (IGT) |  |  |  |
| Fasting | < 6.1(110) | < 6.1(110) | < 7.0(126) |
| And 2 hours after the load | ≥6.7(120) | ≥7.8(140) | ≥7.8(140) |
|  | ～< 10.1(180 | ～< 11.1(200) | ～< 11.1(200) |
| Impaired fasting glucose (IFG) |  |  |  |
| Fasting | ≥5.6(100) | ≥5.6(100) | ≥6.1(110) |
|  | ～< 6.1(110) | ～< 6.1(110) | ～< 7.0(126) |
| And 2 hours after the load | < 6.7(120) | < 7.8(140) | < 7.8(140) |
| Normal |  |  |  |
| Fasting | < 5.6(100) | < 5.6(100) | < 6.1(110) |
| 2 hours after the load | < 6.7(120) | < 7.8(140) | < 7.8(140) |

If it is measured by whole blood, it should be measured immediately after blood collection; if it is measured by plasma, it should be centrifuged immediately after blood collection and (or) be placed in 0 ~ 4 ° for storage. But the latter can not prevent blood cells use blood sugar. Therefore, it would be better to separate the plasma immediately.

**Trial procedures (* indicates items observed by TCM hospitals)**

| **Study stage** | **Run-in** | **Treatment period** | | | | | | | | | | | | |
| --- | --- | --- | --- | --- | --- | --- | --- | --- | --- | --- | --- | --- | --- | --- |
| **Number of follow-up** | **1** | **2** | **3** | **4** | **5** | **6** | **7** | **8** | **9** | **10** | **11** | **12** | **13** | **14** |
| **Test time (weeks)** | **-4** | **0** | **4** | **8** | **12** | **16** | **20** | **24** | **28** | **32** | **36** | **40** | **44** | **48** |
|  | | | | | | | | | | | | | | |
| Informed consent | √ |  |  |  |  |  |  |  |  |  |  |  |  |  |
| General Information | √ |  |  |  |  |  |  |  |  |  |  |  |  |  |
| Vital Signs | √ | √ | √ | √ | √ | √ | √ | √ | √ | √ | √ | √ | √ | √ |
| Inclusion/exclusion criteria | √ | √ |  |  |  |  |  |  |  |  |  |  |  |  |
| Randomization |  | √ |  |  |  |  |  |  |  |  |  |  |  |  |
| Blood and urine routine | √ |  |  |  |  |  |  | √ |  |  |  |  |  | √ |
| Liver and kidney function | √ |  |  |  |  |  |  | √ |  |  |  |  |  | √ |
| Twelve-lead ECG |  | √ |  |  |  |  |  | √ |  |  |  |  |  | √ |
| Fasting blood glucose | √ | √ | √ | √ | √ | √ | √ | √ | √ | √ | √ | √ | √ | √ |
| Investigational drug dose adjustment |  |  | √ | √ | √ | √ | √ | √ | √ | √ | √ | √ | √ | √ |
| 2h postprandial blood glucose |  | √ |  |  |  |  |  |  |  |  |  |  |  | √ |
| Fasting insulin # |  | √ |  |  | √ |  |  | √ |  |  | √ |  |  | √ |
| C peptide # |  | √ |  |  | √ |  |  | √ |  |  | √ |  |  | √ |
| HbA1c# | √ | √ |  |  | √ |  |  | √ |  |  | √ |  |  | √ |
| Blood lipid # |  | √ |  |  | √ |  |  | √ |  |  | √ |  |  | √ |
| Inflammatory factors (hsCRP)# |  | √ |  |  | √ |  |  | √ |  |  | √ |  |  | √ |
| Adiponectin # |  | √ |  |  | √ |  |  | √ |  |  | √ |  |  | √ |
| Urinary albumi/creatinine |  | √ |  |  |  |  |  |  |  |  |  |  |  | √ |
| Brachial artery ultrasonography* |  | √ |  |  |  |  |  |  |  |  |  |  |  | √ |
| Nocturia index * |  | √ |  |  |  |  |  | √ |  |  |  |  |  | √ |
| Weight, waist circumference, hip circumference | √ | √ | √ | √ | √ | √ | √ | √ | √ | √ | √ | √ | √ | √ |
| Collect 10ml anticoagulation blood anticoagulated blood |  | √ |  |  |  |  |  |  |  |  |  |  |  |  |
| Collect 10ml urine |  | √ | √ | √ | √ | √ | √ | √ | √ | √ | √ | √ | √ | √ |
| Evaluation of TCM diabetes symptoms | √ | √ | √ | √ | √ | √ | √ | √ | √ | √ | √ | √ | √ | √ |
| hypoglycemia |  |  | √ | √ | √ | √ | √ | √ | √ | √ | √ | √ | √ | √ |
| Adverse events |  | √ | √ | √ | √ | √ | √ | √ | √ | √ | √ | √ | √ | √ |
| Release of metformin | √ |  |  |  |  |  |  |  |  |  |  |  |  |  |
| Release of investigational drug |  | √ | √ | √ | √ | √ | √ | √ | √ | √ | √ | √ | √ |  |
| Recovery of investigational drug |  |  | √ | √ | √ | √ | √ | √ | √ | √ | √ | √ | √ | √ |
| Combined medication |  | √ | √ | √ | √ | √ | √ | √ | √ | √ | √ | √ | √ | √ |

**Notes: Samples for genomics, insulin, C peptide, HbAlc, blood lipids, hsCRP, and adiponectin indicators will be all measured by Central Lab;**

**Contents**

Notice

Trial procedures

Table of Abbreviations

[I. Research background 1](#__RefHeading___Toc296501850)

[II. Trial objectives and goals 5](#__RefHeading___Toc296501851)

[III. Trial endpoints 6](#__RefHeading___Toc296501852)

[IV. The overall design of the trial 7](#__RefHeading___Toc296501853)

[V. Case selection criteria 10](#__RefHeading___Toc296501854)

[VI. Dosage regimens 13](#__RefHeading___Toc296501855)

[VII. Monitoring indicators 16](#__RefHeading___Toc296501856)

[VIII. Evaluation of clinical efficacy and of relevant laboratory test results 19](#__RefHeading___Toc296501857)

[IX. Follow-up plan 20](#__RefHeading___Toc296501858)

[X. Adverse events 21](#__RefHeading___Toc296501859)

[XI. Data Management 24](#__RefHeading___Toc296501860)

[XII. Statistical analysis 25](#__RefHeading___Toc296501861)

[XIII. Ethics requirements 27](#__RefHeading___Toc296501862)

[IVI. Anticipated study objectives 28](#__RefHeading___Toc296501863)

[XV. Expected progress and completion date of the clinical trial 28](#__RefHeading___Toc296501864)

Appendixes

References

**Table of Abbreviations**

| **Abbreviations** | **English name** |
| --- | --- |
| 2DM、T2DM | Type 2 diabetes |
| BMI | Body mass index = weight (kg)/height^2^ (m^2^) |
| FPG | Fasting plasma glucose |
| P2hPG、2hPG | 2h postprandial blood glucose |
| IGT | Impaired glucose tolerance |
| IFG | Impaired fasting glucose, abnormal fasting glucose |
| HbA1c | Glycated haemoglobin Alc |
| ADOPT trial | A Diabetes outcome progression Trial |
| GCK | Glucokinase gene |
| TZDs | Thiazolidinediones (glitazones) |
| Ins | Serum insulin |
| HOMA-IR | Insulin resistance index HOMA-IR = FINS × FPG/22.5 |
| HOMA- IS | Islet β-cell function, insulin secretion index HOMA-IS = 20 × FINS/(FPG-3.5) |
| FINS | Fasting insulin |
| P2hINS | 2h insulin |
| hsCRP | Inflammatory factors, high sensitivity C-reactive protein |

**Evidence-Based Medical Research Protocol of Zhongyi Xiaoke Pills**

## I. Research background

1．Introduction of Chinese Traditional Medicine in treating Type 2 diabetes

Type 2 diabetes accounts for about 95% of the diabetes population, and its causes are complex, involving multiple genetic and environmental factors and their interactions. As the pathogenesis is not very clear so far, so it is very difficult to carry out long term effective treatment for diabetes at present. In big cities, only about 30% of diabetes patients have their blood glucose levels well-controlled, while in the medium and small cities and rural areas, the control rate is even lower. The low control rate is mainly due to:

(1) Currently, although there are effective drugs for the control of diabetes, but as treatment with the drugs cannot be individualized, so they cannot fully exert their greatest therapeutical effects on sensitive individuals;

(2) As the dosage is reduced in some patients due to their side effects, the drugs cannot fully exert their therapeutical effects;

(3) Drug treatment begins too late, which missed the best window phase of the drugs to prevent the progression of diabetes through correction of the pathophysiologic changes of diabetes, resulting in the course of diabetes progression to a stage that is difficult to control.

At present, oral drugs used clinically to control blood sugar mainly include the sulfonylureas, biguanides, and glitazones. The results of ADOPT published recently showed that although glibenclamide monotherapy only maintained the blood glucose well-controlled for a time significantly shorter than metformin and rosiglitazone, and its risk to cause hypoglycemia was significantly increased, but glibenclamide was significantly superior to metformin and rosiglitazone in the cardiovascular safety; compared with the glitazones, glibenclamide caused less weight gain.

As an inexpensive hypoglycemic drug that has been widely used for a long term, the results of ADOPT once again confirmed the cardiovascular safety of glibenclamide. Therefore, it will still be used as the mainstream drug for the treatment of type 2 diabetes and will be more widely used.

Treatment of diabetes with Traditional Chinese Medicine (TCM) has a long history in China. Diabetes was described as “Xiaoke (wasting-thirst) ” in Traditional Chinese Medicine in an ancient Chinese Medical textbook “ Huangdi Nei Jing or The Yellow Emperor’s Classic of Internal Medicine”.

The classic symptoms and signs of “Xiaoke” include sweet urine, dry mouth, thirst, polydipsia, polyorexia, polyphagia, emaciation, and fatigue. In the literature of TCM, it was recorded that the symptoms of “Xiaoke” could be alleviated by TCM. Since “Xiaoke” was described or diagnosed by symptoms and signs rather than by hyperglycemia, TCMs for “Xiaoke” were developed on the basis of treating the symptoms belong to “Xiaoke” but not on lowing blood or urine glucose. Evidence of hypoglycemia effect of TCM was mainly coming from animal studies using extract from a single plant in the formula of classic TCM for Xiaoke. Whether TCM can lower glucose in human being is still inconclusive. Therefore, the roles of TCM in lowing glucose remain to be elucidated.

In modern China, some hypoglycemia medications were compound preparations of Chinese herbs combined with western medicine such as glibenclamide. One of such preparations is Xiaoke Pills. Xiaoke Pills is a compound preparation of Chinese herbs combined with western medicine (glibenclamide) produced by Guangzhou Zhongyi Pharmaceutical Co., Ltd. for the treatment of type 2 diabetes, which has been approved as one of the Protected Traditional Chinese Medicinal Products of P.R.C. Early study results suggest that Xiaoke Pills can nourish kidney-*yin*, replenish *qi*, and promote generation of fluid, which is indicated for diabetes due to deficiency of *qi* and *yin* (type 2 diabetes) manifested as polydipsia, polyphagia, fatigue, shortness of breath, indolent about speaking.

So far, the results of five RCTs compared Xiaoke Pill and glibenclamide were reported in medical journals in China. All studies were open label with study duration between 4 to 8 weeks. Four studies were done in single center ones. All study showed significant improvement in diabetes symptoms in favor of Xiaoke Pill. Instead using absolute changes in glucose and HbA1c, all trails used proportion of glucose or HbA1c below certain levels as the indicators of efficacies. Two 4 week studies comparing HbA1c, fasting glucose and postprandial did not find differences between Xiaoke Pill and glibenclamide. Three 8 week studies comparing fasting glucose found Xiaoke Pill was associated with significant greater improvement in fasting glucose control as compared with glibenclamide. Only one study assessed hypoglycemia event (450 study subjects, two mild hypoglycemia events in Xiaoke pill group, one in glibenclamide group). The poor qualities of these clinical trials make it hard to draw any solid conclusions with regard to efficacy and safety of Xiaoke pill. However, the validity of the design of these studies is questionable, and makes it hard to draw any robust conclusion with regard to safety and efficacy of Xiaoke Pill.

Therefore, in order to better guide the clinical application of Xiaoke Pills containing glibenclamide ingredients, improve the efficacy of medication, reduce the adverse drug reactions, and understand the differences between Xiaoke Pills and pure glibenclamide preparations in clinical efficacy, adverse drug reactions and biological basis, this project intends to explore the effect of genetic, metabolic background as well as their mechanisms in determining the differences in response to Xiaoke Pills and glibenclamide so as to provide basis for the development of personalized therapy plan for different type 2 diabetic patients to improve the efficacy of Xiaoke Pills, and to establish clinical treatment regimen of Xiaoke Pills based on molecular typing.

2. Personalized therapy of diabetes

To understand the basic cause of diabetes is the key for implementation of effective and safe personalized therapy. Recent studies have shown that it can guide the implementation of more effective and safer management of diabetes through molecular typing of diabetic patients. For instance, although diabetes due to glucokinase gene (GCK) mutations manifests as high blood sugar, but this type of diabetes rarely deteriorate, and the blood glucose levels remain only slightly above the level of the diagnostic criteria of diabetes, rarely resulting in complications of diabetes. Long-term follow-up study at abroad has shown that once patients are determined to have this type of diabetes by molecular diagnosis and typing, they can be treated basically as normal people and do not need close follow-up and special treatment. Another example is a recent study showed that hyperglycemia in some patients with previously diagnosed insulin-dependent diabetes (type 1 diabetes) was actually caused by the KCNJ11 gene mutation, this type of diabetes patients may take oral sulfonylureas for glycemic control without the need for insulin therapy, thereby reducing the burden of patients caused by long-term administration of insulin and reduce the risk of hypoglycemia recurring due to insulin injections. Again, among patients with clinically diagnosed type 2 diabetes, about 5-10% of the patients carry the immune markers of type Ⅰ diabetes; this kind of diabetes patients are defined to have “Latent Autoimmune Diabetes in Adults, LADA” because their natural course of disease is distinctly different from typical type Ⅰ diabetes, while in drug treatment, oral sulfonylureas should be avoided to reduce the failure rate of islets β-cells that secrete insulin.

Now it is considered that individual differences in drug effects (therapeutic, toxic and side effects) are mainly decided by the differences in genetic background of individuals in view of drug effects and metabolism. By studying the genetic basis of pharmacodynamics, it may provide the basis for individualized medication guided by genetic information in the future. This can further improve the efficacy of drugs at an overall level of population and reduce the delay in disease condition and unnecessary medical expenses caused by weak therapeutic efficacy in some non-sensitive individuals. It may also find molecular markers related to the side effects and toxicity of drugs to predict the individual’s sensitivity to the drugs, thus to reduce the incidence of toxic and adverse reactions, and the incidence of iatrogenic incidents. Currently, the main purpose of pharmacogenomics research carried out internationally is to understand the reasons why the same drug product has different effects on different people, and to find out the human genetic differences between individuals that lead to differences in efficacy, and to apply the research results in clinical treatment.

Studies have shown that in type 2 diabetes patients in Finland, the diabetes-related PPARγ gene polymorphism is relevant with and the effect of exercise in lowering blood glucose. In addition, a large-scale diabetes prevention study (DPS) completed in this population also found that Leu72Met polymorphism of the blood glucose metabolism-related intestinal hormone growth hormone-releasing peptide (ghrelin) is relevant with the effect of lifestyle in preventing diabetes. A study in Korea found that the GG genotype of adiponectin gene SNP45 was related to the therapeutic efficacy of the oral hypoglycemic agent rosiglitazone. Individuals with type 2 diabetes who carry GG genotype showed poor sensitivity to rosiglitazone therapy. Studies also showed that drug metabolism-related CYP2C9 gene polymorphisms (CYP2C9 * 3) was relevant to the in vivo drug concentration of some insulin secretagogue.

3. The molecular typing of disease, biomarkers, current situation and perspectives of personalized therapy

3.1 Pharmacogenetics study

Pharmacogenetics is a science to study the effect of genetic polymorphism on drug reactions (including drug absorption, metabolism, distribution and excretion, drug safety and tolerability, drug efficacy). Pharmacogenomics is developed on the basis of pharmacogenetics. It is a science based on functional genomics and molecular pharmacology, which applies genomics to investigate the individual differences in drug reactions, demonstrate and elaborate the efficacy as well as the targets, mode of action, toxic and side effects of drugs from the molecular level.

Genetic polymorphism is the basis of pharmacogenomics. Genetic polymorphism of drugs can manifest as the polymorphism of drug metabolism enzymes (affecting drug metabolism, such as cytochrome P_450_), the polymorphism of drug transport proteins (affecting drug absorption, distribution and excretion, such as P-glycoprotein), and the polymorphism of receptors or targets of drugs. The existence of these polymorphisms may lead to individual differences in efficacy and adverse reactions that are related to in vivo drug concentration in various medications.

Pharmacogenomics study is different from the conventional genetic research. Its purpose is not to discover new genes, explore the pathogenesis of the diseases, foresee the risk of disease onset, and diagnose the diseases, but to study the effect of genetic factors on drug efficacy, determine the drug action targets, and investigate the individual diversity in drug reactions from the phenotype to the genotype. The human body has many genes, and a series of mutations may exist in each gene, but the value of any single gene mutation on prediction or treatment of a disease is limited. In contrast, the impact of mutation in a single gene on drug effects is very significant. Therefore, the study of drug effect-related genes has much greater clinical application value than the study of disease-related genes. Pharmacogenomics has closely linked the genetic polymorphism to the individual diversity in drug effect, and makes the findings easier to be applied in clinical practice.

The core of rational medication is individualized medication. Pharmacogenomics will guide the prescription of “genetic prescription” that is suitable for each individual in clinical practice through detection of patients’ genes, e.g., to detect the single nucleotide polymorphism (SNP) in some disease-related genes and drug metabolism-related genes, thus to detect the SNP difference in affected populations who are sensitive or resistant to the specific drug, so that patients can not only achieve the best treatment efficacy, but also can avoid the adverse drug reactions, truly achieving the goal of “personalized therapy”.

In 2005, FDA issued the Pharmacogenomic Data Submissions guidelines for the pharmaceutical companies. This guideline is aimed at urging pharmaceutical companies to necessarily or voluntarily provide the pharmacogenomic data of the drug in accordance with specific circumstances when they submit new drug applications, which aims to promote the progression of more effective new “individualized medication”, and ultimately medicate according to “each individual’s genetics state”, so that the patients can get the best drug effects while facing only minimal risk of adverse drug reactions.

Currently, enzymes genes related to the metabolism of the commonly used hypoglycemic agent metformin mainly include CYP2C11, CYP2D1, CYP3A1/2, AMPK, OCT1 and OCT2, sulfonylureas metabolism-related enzyme genes mainly refer to CYP2C9, some genes related to βcell function, and glitazones metabolism-related enzymes genes mainly include CYP2C8, and CYP3A4.

3.2 Metabolomics

Metabolomics is a discipline developed recently following the genomics and proteomics developed, which is an important component of systems biology. Genomics and proteomics are to respectively explore life activities from the protein level, but in fact many intracellular life activities are associated with metabolites, e.g., cell signaling and energy transfer are all regulated by metabolites. Metabolomics is a discipline to study the collection of all metabolites of metabolome in cells at a certain time point. Genes are closely related to the expression of proteins, while metabolites mostly reflect the environment where the cells are living, which in turn is closely related to the nutritional status of cells, the effect of drugs and environmental pollutants, and other external factors.

Metabolomics is mainly to study the small-molecule metabolites (MW <1000) as the substrates and products of a variety of metabolic pathways. The samples mainly include urine, plasma or serum, saliva, and cell and tissue extracts. The main technical means include nuclear magnetic resonance (NMR), liquid chromatography-mass spectrometry (LC-MS), gas chromatography-mass spectrometry (GC-MS), chromatography (HPLC, GC), etc. Through detecting the spectrogram of a series of samples in combination with chemical pattern recognition methods, it can find the biomarkers that correlate with the pathophysiological state of organisms, gene function, drug toxicity and efficacy. Therefore, metabonomics has very extensive and important perspectives for application in scientific fields including new drug safety evaluation, toxicology, physiology, early diagnosis of grave diseases, personalized therapy, etc.

3.3 Molecular typing techniques

Currently, biochip technology and metabolomics technologies based on genomics, functional genomics and proteomics are applied to analyze and compare the differences in congenital genetic information (SNP), gene expression, types and quantities of proteins, as well as types and quantities of in vivo metabolites between the diseased population and healthy population as well as in the natural course of diseases; moreover, these molecular information will be combined with the biological characteristics, clinical symptoms, treatment response, prognosis and outcome of diseases for comprehensive analysis to establish standards for molecular typing of diseases as well as predictors for predicting the natural course of diseases and treatment monitoring methods, so as to make up the shortage in current clinical routine diagnosis and efficacy evaluation, promote the diagnosis and treatment of the diseases advance into the molecular diagnostics level from the clinical common practice level, and provide basis for personalized therapy of these diseases.

Therefore, the translation of the achievements of disease genetics, metabolomics, and pharmacogenomics studies into clinical medicine will start a new era of “molecular medicine” and “personalized therapy”. The new disease prevention and treatment model on the basis of molecular medicine will enable the individualization of prevention for individuals at high risks and treatment of individuals with diseases.

## II. Trial objectives and goals

**1. Trial objectives**

1.1 To explore the effect and mechanism of genetic, metabolic factors on the differences in response to treatment with Xiaoke Pills and glibenclamide.

1.2 Base on the abovementioned finding, to guide the clinical application of Xiaoke Pills, improve the effectiveness of the drug, and provide basis for individual treatment of different type 2 diabetic patients, and to establish clinical treatment regimens of Xiaoke Pills based on molecular genotyping.

**2. Trial goals**

2.1 To discover biomarkers related to the clinical efficacy of Xiaoke Pills with glibenclamide as a reference.

2.2 To discover biomarkers related to the main side effect, i.e., hypoglycemia and study pathophysiological and clinical features of susceptibility to hypoglycemia.

2.3 To discover the unique efficacy of Xiaoke Pills compared with sulfonylureas, as well as the biomarkers associated with the unique efficacy of Xiaoke Pills.

2.4 To discover biomarkers, pathophysiological and clinical features associated with secondary failure of sulfonylureas. To investigate if Xiaoke Pills can reduce the risk of secondary failure of sulfonylureas as compared with glibenclamide.

2.5 Apply the TCM evaluation indexes to evaluate the efficacy and safety of Xiaoke Pills and glibenclamide tablets from the view of treatment of type 2 diabetes belonging to the syndrome of dual deficiency of qi and yin, and to found the biomarkers associated with above evaluation indexes.

## III. Trial endpoints

**1. Primary endpoint**

The primary endpoint of this study is the change in HbA1c levels at 48 weeks from baseline, compared between the treatment groups.

**Study Termination Criteria**

When fasting blood glucose is found to exceed 126 mg/dl (7.0 mmol/L) for the first time after Xiaoke Pills is administered reaching a dose of 30 pills/day or glibenclamide is administered reaching a dose of 3 tablets/day, if the fasting plasma glucose level is still below 200 mg/dl (11.1 mmol/L), the patient may be observed continuously till the next follow-up. During this period, the patient is told to test blood glucose at home by him/herself, if fasting blood glucose (finger whole blood) exceeds 234 mg/dL (13 mmol/L) on 2 consecutive testing, the patient should withdraw from the study and receive conventional treatment.

**2. Secondary endpoints and indicators**

2.1 Incidence and rate of hypoglycemia;

2.2 General efficacy (FPG, 2hPG, HbAlc) and the efficacy of TCM Syndrome of dual deficiency of *qi* and *yin* ;

2.3 Proportion of patients with HbA1c < 6.5% at 48 week;

2.4 The time to achieve HbA1c <6.5%;

2.4 Changes in islet β-cell function (HOMA-IS = 20 × FINS/(FPG-3.5));

2.5 Changes in insulin resistance levels (HOMA-IR = FINS × FPG/22.5);

2.6 Changes in weight, waist circumference, and hip circumference;

2.7 Changes in lipids levels, insulin levels, inflammatory factors (hsCRP), and adiponectin;

2.8 Metabolomics and pharmacogenetics;

## IV. The overall design of the trial

**1. Sample size**

The sample size of the trial is designed and estimated according to non-inferiority test. The assumption of non-inferiority test is that the Xiaoke pill is not inferior to glibenclamide in terms of reducing HbA1c. Xiaoke Pill was regarded as non-inferior to treatment with Glibenclamide only if, after 48 weeks of treatment, the upper limit of the two-sided 95% CI for the difference in mean HbA1c change was less than 0.4%. A sample size of 400 patients was estimated to provide more than 90% power to test the hypothesis that Xiaoke Pill treatment was non-inferior to treatment with Glibenclamide in each of the study groups, assuming a 20% early discontinuation rate, and an expected inter-patient SD of 1.1%. The sample size estimation is based on being able to provide sufficient amount of information.

According to statistical results of sample size, in order to better guide the clinical application of Xiaoke Pills to improve the effectiveness of medication, this study is plan to enroll 400 non-obese patients with type 2 diabetes who have not received any antidiabetic agents before and have unsatisfactory glycemic control on diet and exercise and 400 patients with type 2 diabetes who have unsatisfactory glycemic control after treatment with metformin alone for over 3 months. 800 patients will be randomly assigned to the treatment groups and control group.

**2. Design of the trial**

This study is a randomized, double blind and double dummy, masculine parallel comparison, multi-center clinical trial.

It is estimated that a total of 1600 type 2 diabetes patients with unsatisfactory glycemic control will participate in preliminary screening, of which 800 patients have not used any hypoglycemic agents and are non-obese (hereinafter referred to patients for initial treatment), and 800 patients have unsatisfactory glycemic control after treatment with metformin alone for over 3 months (hereinafter referred to patients treated with metformin). After determined as eligible through preliminary screening, about 1,000 patients (each 500 of patients for initial treatment and patients treated with metformin) will enter the four-week run-in period. During the run-in period, it will maintain the same normal dose of metformin for patients treated with metformin, in addition to diet and exercise management.

After the end of run-in, those who still meet the inclusion criteria (a total of 800 patients, including each 400 in the initial treatment group and metformin treatment group) will be randomly divided into the test group and control group, i.e., Xiaoke Pills group and glibenclamide group (n = 400 patients). In the glibenclamide group, the initial dose is glibenclamide 1.25 mg (0.5 tablets) with simulator of Xiaoke Pills 5 pills, once daily, and the maximal dose is glibenclamide 7.5 mg/d for oral administration in 3 times; in the Xiaoke Pills group, the initial dose is Xiaoke Pills 5 pills with simulator of glibenclamide tablets 1.25 mg (0.5 tablets), once daily, and the maximal dose is Xiaoke Pills 30 pills/d for oral administration in 3 times. Each subject will be followed up for 48 weeks, and the necessity for dose adjustment will be evaluated every 4 weeks according to fasting blood glucose levels. If the patient has achieved the Study Termination Criteria of the clinical trial, then the trial is terminated and transferred to conventional treatment, while the treatment methods comply with the “China Guidelines for Diabetes Prevention and Treatment”.

**3. Trial procedures**

4 weeks

Control group (glibenclamide)

Run-in period

Test group (Xiaoke pills)

组

48 weeks

Initially treated patients

/Metformin treated patients

Randomization

**4. Randomization**

Biometric experts will use SAS software to randomly divide the subjects who have been determined as eligible through screening in the run-in period into group A or group B, after stratification according to initial treatment and metformin treatment as well as test centers. The specific method of randomization is:

1. Each case to be observed will be given a code like 001-800;
2. Biometric experts will use the SAS statistical software procedure statement of the computer to assign the number of seeds and number of centers, automatically obtaining the random number table; through the random number table, the random number is obtained, which is one-to-one with the case code;
3. Patients are divided into group A and group B according to the ratio of 1:1, and treatment of the two groups will be randomly decided;
4. Corresponding kit is prepared according to the case code and grouping, and the medication number is as the same as the case code;

⑤ When eligible patients enter the test, according to the order of entry, find the kit of the same number as the case code to give treatment.

The treatment of enrolled subjects is only known by the data centers and drug distribution centers, while both the subjects and investigators do not know the situation on grouping.

**5. Preservation of blind codes and regulation on unblinding**

5.1 Preservation of blind codes:

The two-level blind method is adopted for design, the first level is that the corresponding group of each case code is either group A or group B, and the second level is that the corresponding treatment of the two groups is either the test group or control group. The blind codes of the two levels is sealed separately, each in duplicate, which are respectively preserved in the drug clinical trial test base of the team leader’s unit and the sponsor.

5.2 Emergency unblinding:

Each study drug with a code has a corresponding emergency letter, and a paper strip recorded with the type of treatment given to the drug of the code is placed in the emergency letter, so as to be used for unblinding in case of emergency. In case of emergency or serious adverse events when the subjects require emergent rescue, the principal investigator of the center determine whether it needs to open the emergency letter. Once the emergency letter is opened, the case is considered as lost, which will not be included in efficacy analysis, but if there are any adverse reactions, it should be included in the analysis of adverse reactions.

5.3 Regulation on unblinding:

Two times of unblinding is used in this trial. After completion of data entry and inspection of blinded state, the data is locked. The first unblinding is performed by the personnel of Statistics Centre who keeps the blind end, i.e., inform the corresponding group of each case code to biostatistician using the code name A or B for statistical analysis of all data. When the statistical analysis is ended and the statistical report is completed, the second unblinding is performed by the sponsor and the team leader’s unit to announce the treatment of group A and B, and finally complete the clinical trial summary report.

In the process of the clinical trial, if all the blind ends are disclosed or over 20% of the emergency letters are opened and read, this double-blind trail is failed.

**6. Case allocation**

It plans to assign the cases to each research center as follows:

| **Research Center** | **Patients for initial treatment**  **(test group: control group)** | **Patients treated with metformin**  **(test group: control group)** |
| --- | --- | --- |
| China Academy of Chinese Medicial Sciences Guang’anmen Hospital | 16:16 | 16:16 |
| Shanghai University of Traditional Chinese Medicine Yueyang Hospital of Integrated Traditional Chinese and Western Medicine | 16:16 | 16:16 |
| The First Affiliated Hospital of Guangzhou University of Traditional Chinese Medicine | 16:16 | 16:16 |
| Beijing University of Traditional Chinese Medicine Dongfang Hospital | 10:10 | 10:10 |
| The Third Affiliated Hospital of Peking University of Traditional Chinese and Western Medicine | 16:16 | 16:16 |
| Peking University People’s Hospital | 16:16 | 8:8 |
| Peking University First Hospital | 6:6 | 6:6 |
| Shanghai Jiaotong University Ruijin Hospital | 6:6 | —— |
| Zhongshan University Sun Yai-sen Memorial Hospital | 12:12 | 6:6 |
| Sichuan University West China Hospital | 12:12 | 18:18 |
| General Hospital of PLA Second Artillery | 8:8 | 8:8 |
| The Central Hospital of China Aerospace Corporation | 8:8 | 16:16 |
| The First Affiliated Hospital of Chongqing Medical University | 14:14 | 14:14 |
| The First Hospital of Hebei Medical University | 14:14 | 20:20 |
| China Meitan General Hospital | 12:12 | 12:12 |
| The Second Xiangya Hospital of Central South University | 6:6 | 6:6 |
| The Second Affiliated Hospital of Chongqing Medical University | 6:6 | 6:6 |
| Nanjing Hospital of Traditional Chinese Medicine | 6:6 | 6:6 |
| **Total** | 200:200 | 200:200 |

## V. Case selection criteria

**1. Inclusion criteria**

1.1 Patients with type 2 diabetes;

1.2 Belonging to TCM Syndrome of dual deficiency of *qi* and *yin*;

1.3 Aged 21～70 years;

1.4 Patients for initial treatment: Patients who have not received any hypoglycemic agent and are non-obese,, with body mass index (BMI) within 18kg/m^2^ ~ 28 kg/m^2^ (not included);

1.5 Patients treated with metformin: Patients have received treatment with metformin (immediate-release) at a stable dose ≥ 750 mg/day for at least 3 months before screening, with body mass index (BMI) within 18kg/m^2^ ~ 35 kg/m^2^ (not included);

1.6 Body weight remains constant (body weight fluctuates by less than 10% within at least 3 months before screening);

1.7 Poor glycemic control, fasting blood glucose greater than 126 mg/dl (7.0 mmol/L) and HbA1c >7.0% (preliminary screening), fasting blood glucose greater than 126 mg/dl (7.0 mmol/L) (after run-in);

1.8 Patients still meet the above criteria (without considering HbA1c) after a 4-week run-in period.

**2. Exclusion criteria**

2.1 Personnel of the research base who have a direct relationship with this study, as well as their close relatives. Close relatives refer to the spouse, parent, child, brother or sister, whether due to blood relationship or legal adoption.

2.2 Employees of Guangzhou Zhongyi Pharmaceutical Co., Ltd. (including staff, temporary contractual workers or designated personnel participate in this study.)

2.3 Have participated in this study in the past or other studies using sulfonylureas.

2.4 Participated in intervention medical, surgical or pharmacological research (research that gives study drugs, medical or surgical therapy) within 30 days before screening. This standard includes drugs that have not been approved by drug administration departments when entering the study.

2.5 Women of childbearing age who meet the following conditions (not yet received surgical contraception and are at the stage from menarche to 1 year after menopause):

1) Lactating women.

2) Positive pregnancy test results at the enrollment.

3) Plan to get pregnant during the research.

4) Did not take effective birth control measures within 3 months before screening.

5) Patients do not agree to continue the use of effective birth control means in the process of the trial, as determined by the investigators.

2.6 Fasting blood glucose ≥ 13 mmol/L, or HbA1c ≥ 11%.

2.7 Have more than 3 episodes of severe hypoglycemia within 6 months before the screening.

2.8 Those that who are allergic to sulfonylureas or their ingredients.

2.9 With contraindications of using metformin or sulfonylureas according to the drug package insert.

2.10 Had received treatment with exogenous insulin for more than 1 week within 3 months before screening.

2.11 Those who have a history of clinically significant heart disease within 1 year before entering the study, or with active heart diseases including myocardial infarction, severe arrhythmia, unstable angina, moderate or severe heart failure (New York Heart Society cardiac function grading: class III or IV), coronary artery bypass surgery or reconstructive vascular operation, or those who are estimated to require coronary artery bypass surgery or reconstructive vascular operation during the study.

2.12 Patients who have a history of kidney transplant or are currently receiving dialysis treatment or with serum creatinine levels reaching the upper limit of normal value.

2.13 With obvious clinical signs or symptoms of liver disease, acute or chronic hepatitis, or alanine aminotransferase (ALT or AST) greater than 2.5 times the upper limit of normal value.

2.14 Suffering from hemoglobin disease or chronic anemia.

2.15 Suffering from active proliferative kidney disease.

2.16 Patients who are receiving any of the following medications are to be excluded:

1) Have used thiazolidinediones (TZD) within 3 months before screening.

2) Have used insulin secretagogue (sulfonylureas or nateglinide) within 1 month before screening.

3) Have used α-glucosidase inhibitors (such as miglitol or acarbose) within 1 month before screening.

4) Drugs that directly affect the gastrointestinal motility including metoclopramide and cisapride, and long-term use of macrolide antibiotics, but not limited to these drugs.

5) Systemic glucocorticoid treatment (not including local or inhaled medication) by oral administration, intravenous (IV) or intramuscular (IM) injection on a regular basis (more than 1 month) or within 1 month before screening.

2.17 Suffering from active or untreated malignancy, or malignancy in clinical remission less than 5 years (except for basal cell carcinoma or cutaneous squamous cell carcinoma, cervical carcinoma in situ, or prostate carcinoma in situ).

2.18 With a history of organ transplantation.

2.19 With a present or past history of drug or alcohol abuse that may lead to poor compliance of subjects as determined by the investigators.

2.20 For any other reason, the investigators consider it inappropriate to participate in this study.

**3. Withdrawal criteria**

3.1 After inclusion, the patient is found to not meet the inclusion criteria or meet the exclusion criteria.

3.2 Patients have not received any medication after inclusion for various reasons.

3.3 Patients without any record.

**4. Loss criteria**

4.1 Definition: All subjects who have completed the informed consent, been determined as eligible through screening in the run-in period and have been randomized into the trial, are considered as loss cases as long as if they do not complete the observational period specified in the protocol, no matter at any time and due to any reason.

4.2 Treatment of loss cases

4.2.1 When the subjects are lost, the investigators should contact the subjects as far as possible to inquire about the reasons, record the last medication time, and complete the evaluation items that can be completed.

4.2.2 When the subjects withdraw from the trial due to allergic reactions, adverse reactions, or treatment failure, the investigators should take appropriate therapeutic measures according to the actual situation of the subjects.

**5. Termination (withdrawal)**

In the following situations, the study will be terminated for the patients, but the patients will be included in the FAS statistics:

5.1 If the female patient gets pregnant (violation to the protocol).

5.2 If the patient has not used the study drugs (including metformin) in accordance with therequirements of the protocol for more than 7 consecutive days.

5.3 If the patient has used insulin for more than 7 days, or month than 7 days within 1 month.

5.4 If the patient has used combined therapy with the drugs that should be excluded for more than 7 days.

5.5 If the patient’s (finger whole blood) fasting blood glucose has been greater than 234 mg/dL (13 mmol/L) for 3 consecutive times (lack of efficacy).

5.6 Serious adverse events occur.

5.7 If the patient has recurrent hypoglycemia (more than 2 episodes of hypoglycemia per week in 4 consecutive weeks), especially severe hypoglycemia (more than 2 episodes of severe hypoglycemia within 30 days), the investigator must consider terminating the trial for the patient.

If the investigators determine the patients should withdraw from the trail because of serious adverse events or significantly increased clinical laboratory parameters, appropriate measures should be taken immediately and the principal of the study should be notified.

The investigators terminate the trial or terminate the patients to participate in the trial for any reason; those patients under early termination will continue to carry out the visit procedures for early termination as specified in the protocol procedures.

**6. Subject selection procedures**

Type 2 diabetes patients of initial treatment or irresponsive to metformin therapy who have visited the units participating in the study for a long term, if determined to meet the inclusion criteria through routine examination, they can enter the run-in period. Before screening, it should inform the patients the content, benefits and possible risks of the clinical trial in accordance with the principles of GCP, only when the informed consent is obtained from the patients with their signature, it can perform screening on the patients and let the patients enter the run-in period.

## VI. Dosage regimens

**1. Study drugs**

**Investigational drug**: Xiaoke Pills (each pill contains glibenclamide 0.25mg + Chinese herbs), produced by Guangzhou Zhongyi Pharmaceutical Co, Approval Document Number: SFDA (approved) Z44020045, production batch number: K03300, shelf life: 3 years.

**Control drug:** glibenclamide tablets, 2.5 mg/tablet, produced by Tianjin Pacific Pharmaceutical Co., Ltd., Approval Document Number: SFDA (approved) H12020790, production batch number: 070708, shelf life: 3 years.

**Simulators:** The simulator of Xiaoke Pills and simulator of glibenclamide tablets are prepared in line with the requirements for placebo preparation, which are pills or tablets not containing any active ingredient.

**Basic medication** (patients treated with metformin): metformin hydrochloride tablets, 0.25 g/tablet, purchased by the Guangzhou Zhongyi Pharmaceutical Co., Ltd., Approval Document Number: SFDA (approved) H11020976, shelf life: 3 years.

In order to ensure the implementation of the blindmethod, the study drugs of the trial will be divided into No.1 medication and No.2 medication. No.1 medication includes Xiaoke Pills and simulator of Xiaoke Pills, while No.2 medication includes glibenclamide tablets and simulator of glibenclamide tablets.

**2. Drug packaging**

Drugs to be used in the test group and control group are in identical packaging, and their labels shall be printed with the contents including the actions and indications, usage and dosage, lot number, drug suppliers, drug code, etc., and marked with “drugs for clinical trial” and “only for clinical research”. The bottle, box and label of the investigational drug, control drug and simulators are identical.

No.1 medication and No.2 medication are respectively packaged in two bottles, determined by calculation after dose adjustment. Medications for each visit cycle should be prepared according to the maximal doses that may be administered. Medications for 48 weeks are packaged in a big package. Blind coding is performed according to the random allocation table, and each big package is marked with a drug code from 1-800.

**3. Drug distribution**

Sub-packed kits are sent to each test center according to their randomly assigned number of the test center, along with the corresponding emergency letter, for multi-center clinical trial.

**4. Medication method**

**Study group:**

No. 1 medication: Xiaoke Pills, the initial dose of 5 pills, once daily, p.o.; the maximal daily dose of 30 pills, 3 times daily, p.o.

No. 2 medication: Simulator of glibenclamide tablets, the initial dose of 0.5 (1.25 mg) tablet/day, p.o.; the maximal daily dose of 3 (7.5 mg) tablets/day, 3 times daily, p.o.

**Control group:**

No. 1 medication: Simulator of Xiaoke Pills, the initial dose of 5 pills, once daily, p.o.; the maximal daily dose of 30 pills, 3 times daily, p.o.

No. 2 medication: Glibenclamide tablets, the initial dose of 0.5 (1.25 mg) tablet/day p.o.; the maximal daily dose of 3 (7.5 mg) tablets/day, 3 times daily, p.o.

In addition to the above study drugs, patients treated with Metformin, either in the test group or the control group should also take metformin tablets orally at a dose the same as that at enrollment, while the dose remain the same throughout the trial.

Medication period: 48 weeks.

**5. Dose adjustment**

The dose of the study drugs are to be adjusted every 4 weeks according to fasting blood glucose test results. Specific methods are as follows:

For patients with fasting plasma glucose 4.4-7.0 mmol/L, the dose remains the same.

For patients with fasting plasma glucose > 7.0 mmol/L, add 5 pills to the dose of the No. 1 medication and 0.5 tablet to the dose of No.2 medication.

For patients with fasting blood glucose < 4.4 mmol/L, the dose of the No. 1 medication is reduced by 5 pills and the dose of the No.2 medication is reduced by 0.5 tablet.

Medication times:

1. When the dose is 5 pills of No. 1 medication or 0.5 tablet of No.2 medication, it should be taken orally at one time before breakfast.
2. When the dose is 10 pills of No. 1 medication or 1.5 tablets of No.2 medication, it should take 10 pills or 1 tablet orally before lunch.
3. When the dose is 15 pills of No. 1 medication or 1.5 tablets of No.2 medication, it should be divided into two portions, take 5 pills or 0.5 tablet orally before breakfast and take 10 pills or 1 tablet orally before lunch.
4. When the dose is 20 pills of No. 1 medication or 2 tablets of No.2 medication, it should be divided into two portions, take 10 pills or 1 tablet orally before breakfast and take 10 pills or 1 tablet orally before lunch.
5. When the dose is 25 pills of No. 1 medication or 2.5 tablets of No.2 medication, it should be divided into 3 portions, take 10 pills or 1 tablet orally before breakfast, 10 pills or 1 tablet orally before lunch, and 5 pills or 0.5 tablet orally before supper.
6. When the dose is 30 pills of No. 1 medication or 3 tablets of No.2 medication, it should be divided into 3 portions, take 10 pills or 1 tablet orally before breakfast, 10 pills or 1 tablet orally before lunch, and 10 pills or 1 tablet orally before supper.

**6. Drug inventory and preservation**

The number, shipment, delivery, transition, storage, distribution, recycling, and destruction of study drugs should all be recorded. The records are preserved as the clinical trial documents.

After arrived at each clinical trial center, all study drugs should be submitted to designated personnel and managed at the designated place that meets the requirements of drug storage, and released by designated personnel do not participate in the clinical trial.

Trail managers should regularly check the quality of the drugs.

It only allows the investigators to use the study drugs, and the investigators must ensure that all drugs are only used in subjects enrolled in the clinical trial, and are used strictly in accordance with the clinical trial protocol.

The investigators should recover the remaining drugs at each return visit. After the clinical trial is completed, designated personnel from the sponsor should recover all the remaining study drugs in time.

**7. Combination therapy**

During the trial, the subjects should not use other Chinese herbs or western medicine or other therapies of hypoglycemic effects. For original or newly occurred concomitant diseases (e.g., hypertension) that require medication, the combination therapy should be recorded in details in the combination therapy registration form.

## VII. Monitoring indicators

**1. Conventional indicators**

Blood and urine routine tests, liver and kidney function, blood glucose, insulin, C peptide, glycosylated hemoglobin (HbA1c), blood lipids, inflammatory factors (hsCRP), adiponectin, ECG, weight, blood pressure;

Cardiovascular complications: angina pectoris, myocardial infarction, death. The occurrence of other chronic complications of diabetes (e.g., microalbuminuria).

The incidence of hypoglycemia and other clinical adverse events (patients test their blood glucose at home using glucose meter and record the test results in the blood glucose record card.)

Score of TCM symptoms of diabetes and Pattern of dual deficiency of Qi and Yin: including the main symptoms, secondary symptoms.

Select 100 patients for brachial artery ultrasonography to evaluate whether the Xiaoke Pills has the effects on blood vessel endothelial function.

**2. Pharmacogenetics study**

2.1`Collect 10 ml of peripheral blood sample from the patients, and use phenol-chloroform method to extract genomic DNA.

2.2 Use high-throughput SNP technologies to detect the gene polymorphism of the major targets of glyburide and drug metabolism-related proteins, including sulfonylurea receptor, potassium channel protein Kir6.2, genes associated with βcell function, CYP2C9. Use the above symbolic or “node” protein or molecule and the key molecules of the action and metabolic pathways of biguanides and sulfonylureas as candidate genes to make SNPs biochip, use technologies of genomic detection and analysis platform to detect these SNPs sites, analyze the interactions between SNPs, to establish the correlation between different drug responsiveness and SNPs, so as to identify the SNPs sites that can guide personalized therapy in clinical practice.

**3. Metabonomics study**

Collect the urine samples of baseline and follow-up at each stage of the trial for testing and analysis of the metabolites. Establish and apply high-throughput, hypersensitized metabolomics detection and analysis technologies, including electron paramagnetic resonance spectrometer, LC and GC-MS as well as automatic biochemical analyzer, combined with laser copolymer fluorescence microscopy and other molecular cytobiology and immunology technologies for testing and analysis of the clinical samples of these populations, in particular the trace and rapid decayed metabolic regulation signaling molecules, metabolites of glibenclamide and the Chinese herbal ingredients in Xiaoke Pills; through qualitative and quantitative analysis, identify the metabolic molecules of specific abnormalities (high or low) to obtain the analysis spectrum of related metabolic molecular, so as to determine the most representative and original corresponding indicators that can accurately reflect the therapeutic response.

**Appendix 1:**

**Score of TCM symptoms of diabetes**

(modified from “guiding principle for clinical study of new Chinese medicine treating diabetes” recorded in the book of *Guiding Principles for Clinical Study of New Chinese Medicinal*, Chinese Medical Science and Technology Publishing House, May, 2002, the first edition)

**Main symptoms:** dry throat and mouth, lack of strength.

**Secondary symptoms:** polyphagia, polydipsia, shortness of breath, vexing heat in the chest, palms and soles, palpitations, insomnia, reddish urine, and constipation

| **symptoms** | **Slight（1 point）** | **moderate（2 points）** | **severe（3 points）** |
| --- | --- | --- | --- |
| Dry throat and mouth | Slightly | Occasionally | obviously |
| Fatigue | Can not stand hard work | Able to do light work | Barely for daily activity |
| Polyphagia and easily hungry | Obviously hungry | Unable to stand hungry before meal | Unable to stand hungry and easily accompanied with hypoglycemia |
| Thirsty for drink | Slightly increased of drinking water | Increased amount of drinking water | Double increased of drinking water |
| Short of breath, lazy to talk | Short of breath after laboring | Short of breath after normal activities | Lazy to talk, short of breath all the time |
| Vexation | Occasionally occurred | Vexation | Vexation and sleeplessness |
| Feverish palms and soles | Feverish palms and soles | Feverish palms and soles, like uncovering coat | Like holding cold objects |
| Palpitation | Occasionally occurred | Often occurred, lasting short time | Often occurred, lasting long time |
| Insomnia | Short of sleep and easily wake up | Hard to sleep, and easily wake up | Hard to sleep for the whole night |
| Constipation | Dry stool and hardly defecate | Dry stool and one defecation in 2-3 days | Dry stool and one defecation in more than 3 days |

Note: Score as “0” if there are no symptoms

## VIII. Evaluation of clinical efficacy and of relevant laboratory test results

1. The proportion of patients reaching the endpoint at 3 months, 6 months, and 9 months.

2. FPG, 2hPG, HbAlc.

3. The percentage of patients with HbA1c reaching the standard (6.5%) at 3 months, 6 months, and 9 months.

4. The incidence of hypoglycemia and other complications of diabetes.

6. Laboratory indicators: changes in blood lipid levels, insulin levels, C-peptide level, inflammatory factors (hsCRP), adiponectin.

7. Changes in islet β-cell function (HOMA-IS = 20 × FINS/(FPG-3.5)).

8. Changes in the levels of insulin resistance (HOMA-IR = FINS × FPG/22.5).

9. Genomic and metabolomic studies.

Analyze a large number of SNP and metabolic data to reveal the major genetic andmetabolic that impact the drug efficacy.

## IX. Follow-up plan

A 4-weeks run-in period, 13 times of followed up every 4 weeks after randomization for a total of 48 weeks.

**1. Preliminary screening before the run-in period**

1.1 The patients sign the informed consent and accept the screening.

1.2 The history and diagnosis: history, previously extent underlying diseases, combined medications, Western medical diagnosis and TCM pattern.

1.3 Physical Examination: To measure the height, weight, waist circumference, hip circumference, and vital signs.

1.4 Laboratory indicators: blood and urine routine tests, liver and kidney function tests, fasting blood glucose, detection of HbA1c.

1.5 If all the above items meet the case selection criteria, the patient will be assigned to screening number, and enter a 4-week run-in period.

**2. Baseline inspection items**

2.1 After the end of the run-in period, if all the items still meet the case selection criteria, patients will be randomly assigned to a case number, which remains unchanged during the entire course of the study.

2.2 Carry out the fasting plasma glucose, standard postprandial 2h plasma glucose, HbA1c, plasma insulin, blood C-peptide, inflammatory factors (hsCRP), lipids, adiponectin, urinary albumin-to-creatinine ratio, 12-lead ECG examinations.

2.3 Collect 10 ml of anticoagulated blood for extraction of DNA, and 10 ml of urine for metabonomic study.

2.4 Evaluation of TCM symptoms (TCM Hospitals).

2.5 Distribution of study drugs.

2.6 Additional examinations to be taken in TCM Hospitals: 100 cases to receive the brachial artery color Doppler ultrasonography; nocturia index (nocturia volume/24-hour urine volume, daily urine: urine volume from 8:00 a.m. to 8:00 p.m., nocturia: urine volume from 8:00 p.m. to 8:00 a.m.)

**3. Items to be followed-up at every 4 weeks**

3.1 Evaluation of TCM patterns (TCM Hospitals) and the incidence of hypoglycemia in the past 4 weeks;

3.2 Fasting plasma glucose;

3.3 Weight, waist circumference, hip circumference;

3.4 Collect 10ml urine for metabonomics study.

**4. Items to be followed-up every 12 weeks**

4.1 fasting insulin, C peptide, glycosylated hemoglobin (HbA1c);

4.2 lipids, inflammatory factors (hsCRP), adiponectin;

**5. Items to be tested at the** 24^th^ **week**

5.1 Blood and urine routine tests, liver and kidney function tests;

5.2 nocturia index (nocturia volume/24-hour urine volume);

5.3 12-lead ECG.

**6. Items to be tested at the 48^th^ week**

Standard postprandial 2-hour blood glucose, urinary albumin-to-creatinine ratio, blood and urine routine tests, liver and kidney function tests, 12-lead ECG, blood lipids, C-peptide, insulin, inflammatory factors (hsCRP), adiponectin; 100 cases to receive additional examination of brachial artery color Doppler ultrasonography in TCM Hospitals, patients will undergo willundergo the brachial artery color Doppler ultrasonography once again, nocturia index (nocturia volume/24-hour urine volume) will also be obtained .

Items to be measured at Central laboratory include HbA1c, C-peptide, insulin, blood lipids, C-peptide, insulin, inflammatory factors (hsCRP), adiponectin. Intravenous blood for measurement will be drown, processed according to SOP and shipped to the central laboratory.

## X. Adverse events

**1. Definition**

1.1 Adverse Events: refer to adverse medical events occur after patients or subjects of clinical trials treated with a drug, which do not necessarily have a causal relationship with the treatment.

1.2 Serious adverse events: events occurred during clinical trials requiring hospitalization, prolonged length of hospital stay, disability, affecting the work ability, endangering the life or leading to death, resulting in congenital malformations.

1.3 Adverse drug reactions: refer to harmful, unexpected reactions which have a causal relationship with the application of the drug occurred during the process of normal application of a drug at a dose in line with the requirements. In a clinical trial of a new drug or new usage of a drug, when the therapeutic dose has not been established, all the harmful, unexpected reactions which have a causal relationship with the application of the drug should be considered as adverse drug reactions.

1.4 Hypoglycemia: patients with hypoglycemia will record the episodes of hypoglycemia, blood glucose levels, concomitant symptoms and the treatment in the study diary card provided by the sponsor.

1.4.1 The episode of hypoglycemia is defined as the patient feel that he/she has the signs or symptoms associated with hypoglycemia at any time, or with blood glucose <63 mg/dL (3.5 mmol/L) [IFCC  plasma:<68 mg/dL (3.8 mmol/L))], even if not accompanied by symptoms, signs or not requiring treatment.

1.4.2 Severe hypoglycemia is defined as the patient has onset of symptoms related to hypoglycemia and need help from others, with blood glucose < 50 mg/dL (2.8 mmol/L) [IFCC plasma: <55 mg/dL (3.0 mmol/L) ] or immediately recovered after oral administration of carbohydrate or glucagon or intravenous injection of glucose.

1.4.3 The definition on blood glucose levels of nocturnal hypoglycemia is similar (for example: hypoglycemia is <63 mg/dL, severe hypoglycemia <50 mg/dL), which is according to the adverse event reports and blood glucose levels of the patients. Nocturnal hypoglycemia is defined as the event that occurs before the 1st meal (e.g. breakfast) after wake up from falling sleep.

**Classification of hypoglycemic reactions:**

| **Classification** | **Clinical manifestation** | **Treatment** |
| --- | --- | --- |
| Hypoglycemia | Tachycardia, palpitations, trembling, sweating, hunger, dizziness, blurred vision, impaired concentration, behavior disorders, etc. Patients do not need assistance from others, and can take the initiative to seek treatment. Or blood glucose <63 mg/dL (3.5 mmol/L) [IFCC plasma :< 68 mg/dL (3.8 mmol/L)], even if not accompanied by symptoms, signs or not requiring treatment. | Rapid remission after eating carbohydrates |
| Severe hypoglycemia | Hypoglycemia leads to manifestations such as coma, seizures or nervous system damage, and the patient cannot take the initiative to require treatment or seek help. Accompanied by blood glucose < 50 mg/dL (2.8 mmol/L) [IFCC plasma: < 55 mg/dL (3.0 mmol/L)] | Requiring intravenous bolus injection of glucose or glucagon injection for emergency treatment |
| Nocturnal hypoglycemia | Nocturnal hypoglycemia refers to the event that occurs before the 1st meal (e.g. breakfast) after wake up from falling sleep, presenting with clinical manifestations and blood glucose values of hypoglycemia or severe hypoglycemia | Mild patients should take food, while severe patients need intravenous injection of glucose |

**2. Observation and treatment of adverse events**

Observation and record: Investigators should carefully observe any adverse events occurred in the subjects during the clinical study, require the subjects accurately reflect the changes in condition after medication, and avoid the inductive questions. When observing the therapeutic effect, it should also pay attention to observe unexpected toxicity and side effects (including symptoms, signs and laboratory tests). Adverse events should be recorded in the CRF no matter whether or not associated with the investigational drug, including the occurrence time of adverse reactions, symptoms, signs, degree, duration, laboratory indexes, processing methods, course, results, and follow-up time, and status on combined therapy should be recorded in details in order to analyze the correlation between adverse events and the investigational drug, and the record should be signed and dated.

Medical treatment of subjects: When adverse reactions are found, investigators can take the necessary treatment measures to according to the disease condition, e.g., to adjust the dose, temporarily discontinue the medication, etc., and decide whether to terminate the trial. When serious adverse events occur, units responsible for carrying out the study should take the necessary treatment measures immediately to protect the safety of subjects.

**3. Classification of adverse reactions**

**Mild:** tolerable to the subjects, not affecting the treatment, requiring no special treatment, not affecting the health of subjects.

**Moderate:** unbearable to the subjects, requiring drug withdrawal or special treatment, directly affecting the health of subjects.

**Severe:** life-threatening to the subjects, leading to death or disability, requiring immediate withdrawal or emergency treatment.

**4. Evaluation of correlation between adverse events and the investigational drug**

In accordance with the *Regulation on Reporting and Monitoring of Adverse Drug Reactions*” released by State Food and Drug Administration of China on March 4, 2004, the investigators should carry out a comprehensive analysis according to the specific status on the adverse events occurred in the subject, and the subject’s past medical history, concomitant diseases as well as concomitant medication to determine the relationship between adverse events and the drug. Adverse events determined as definitely relevant, probably relevant, and possibly relevant are considered as adverse drug reactions for statistics of the incidence of adverse reactions.

4.1 Definitely relevant to the investigational drug

There is evidence of using the investigational drug; the occurrence of adverse events and the use of the investigational drug are in chronological order that is reliable; using the investigational drug to explain the cause of the adverse events is more reasonable than using other reasons; the withdrawal reaction is positive; the repeated medication tests are positive; the pattern of adverse events is consistent with the previous understanding of this drug or this kind of drugs.

4.2 Probably relevant to the investigational drug

There is evidence for using the investigational drug; the occurrence of adverse events and the use of the investigational drug are in chronological order that is reliable; using the investigational drug to explain the cause of the adverse events is more reasonable than using other reasons; the withdrawal reaction is positive.

4.3 Possibly relevant to the investigational drug

There is evidence for using the investigational drug; the occurrence of adverse events and the use of the investigational drug are in chronological order that is reliable; the adverse events may be caused by the investigational drug or may be caused by other reasons, the withdrawal reaction is positive.

4.4 Possibly irrelevant to the investigational drug

There is evidence for using the investigational drug; the adverse events may be caused by other reasons, the withdrawal reaction is negative or equivocal; the repeated medication tests are negative or equivocal..

4.5 To be evaluated

The emergence of adverse events and use of the investigational drug are in a chronological order that is unreliable;

4.6 Unable to be evaluated

Patients did not use the investigational drug; or there are other significant reasons that can lead to adverse events.

**5. Serious adverse events and reporting**

For any serious adverse events occurred during the trial, regardless of whether related to the investigational drug or not, the investigators should give timely rescue and treatment, and report to the Ethics Committee of the units responsible for the clinical trial and the sponsor within 24 hours, the sponsor and the investigators should promptly research the serious adverse events occurred, and take the necessary measures to ensure the safety and interests of the subjects, and report to the drug administrative department and health authority in a timely manner, simultaneously report to other researchers of clinical trials involving in the same drug. For serious adverse events that are determined as related to the investigational drug, the sponsor should bear the costs of rescue and treatment of patients and provide the corresponding economic compensation.

Investigators must fill out the “Serious Adverse Event Report Form”, and should record in the original data that when and in what way the serious adverse events occurred, whom they are reported. The sponsor should ensure the reporting procedures that meet the requirements of all laws and regulations.

| Unit | Contact | Tel |
| --- | --- | --- |
| Peking University People's Hospital | JI Linong | 010-88325578 |
| Guangzhou Zhongyi Pharmaceutical Co., Ltd. | Deng Huimin | 13922410616 |
| Guangzhou Boji Clinical Research Center of New Drugs | Wang Shuai-shuai | 13430227886、020-38473208 |

**6. Follow-up**

All adverse events should be followed up till they turn normal or basically normal, so as to ensure the safety of the subjects. According to the severity of adverse reactions, it can select hospitalization, outpatient visit, home visit, telephone, communication and other forms as the way of follow-up.

## XI. Data Management

1. Requirements for filling in data by the investigators

The investigators should ensure that the data is true, accurate, complete, timely and legally recorded in the case report form.

1.1 For patients who enter the trial after completion of the informed consent and determined as eligible through screening, it should carefully and detailedly record all the information that have been observed in the case report forms, without blank entry or omissions.

1.2 It should verify that all the data in the case report forms are completely consistent with the data in the original medical records.

1.3 If it is to make any correction on the case report form that is considered as the original data, it can only use scribing and sidenotes while signed and dated by the investigator.

1.4 The original laboratory test sheet (or a copy of the original laboratory test sheet) should be pasted to the corresponding place in the original medical records.

1.5 Data that is significantly higher or lower, or out of the range that can be accepted in clinical practice, should be verified or reviewed, and necessary explanation should be made by the investigators.

2. Requirements for data auditing by auditors

2.1 Auditors should regularly visit each test center during the trial check the informed consent, screening and inclusion of subjects.

2.2 Ensure that all case report forms are correctly completed and consistent with the original data.

2.3 All errors or omissions have been corrected or noted, signed and dated by the investigators.

2.4 Confirm and record the dose modification, alteration of treatment, combined medication, intercurrent diseases.

2.5 Verify that all the withdrawal and loss of visit of subjects are stated in Case Report Form.

2.6 Verify that all adverse events have been documented, serious adverse events have been reported within the specified time and documented.

2.7 Verify whether the study drugs supplied, stored, distributed, and recovered in accordance with relevant provisions, and make the corresponding records.

3. Database creation and data entry

3.1 Database creation: establish a database system specifically for the trial according to the items in the Case Report Form.

3.2 Data entry: data synchronization entry is made by the data administrator, using two-time entry method.

3.3 Data verification: check each item of the data; for inconsistent values reported, check the original questionnaire one by one, and make corrections.

3.4 If the data administrators have questions in the process of data entry, they may fill out the “data question listing” and return it to the auditors, the investigators will answer the questions in written and sign, and return it to the data administrator. “Data question listing “must be properly preserved.

## XII. Statistical analysis

**1. General considerations**

The efficacy analysis will be based on intention to treat (ITT) population, with supportive analysis with per protocol set (PPS). The PPS includes all randomized patients who have received treatment with study drugs for 48 weeks without violation to inclusion/exclusion criteria or criteria for termination of the study. The ITT population includes all randomized patients receiving at least one dose of study drugs. The safety analysis will be based on ITT population.

For efficacy analyses, changes in the primary and secondary study parameters at 48 week from baseline will be measured, and the inference on the possible non-inferiority will be based on the upper limit of the 95% confidence intervals of the difference in mean changes at 48 week. The incidence and average rate of hypoglycemia will be analyzed using appropriate statistical methods.

A separate statistical analysis plan will be developed in due course detailing all aspects of data analysis. Appropriate exploratory analyses will be conducted, as deemed appropriate.

**2. Treatment of patients**

It will list the main reason for termination of the study by the patients, and will list the incidence and percentage of all patients by treatment groups and visit time. Fisher’s exact test will be used for comparison between treatment groups.

**3. Patients’ features**

Categorical variables (such as sex and ethnicity) will be summarized using the frequency and percentage. Continuous variables (such as BMI and baseline HbA1c) will be summarized using mean, standard deviation, and limit values (minimum and maximum).

**4. Combined medication**

It will list the combined medication on all randomized patients, which will be summarized by treatment groups.

**5. Treatment compliance**

In each visit, the investigators will evaluate patients’ compliance based on glycemic blood control of patients, compliance with visit time, completion of patient diaries, and any indicators considered as necessary by the investigators. It will not collect specific research data for the analysis of treatment compliance.

**6. Adverse events**

Adverse events will be classified according to Medical Dictionary for Regulatory Activities (MedDRA). All adverse events will be listed based on the patient code, visit time, the preferred name, the treatment group, severity, and the relationship with the study drug s. Adverse events will be summarized by number and proportion, and will be compared between the treatment groups using appropriate statistical tests(s).

**7. Analysis of laboratory and vital signs**

Assessment of laboratory classification indexes (clinical biochemistry and blood routine test) will mainly observe the proportion of patients with test results out of the range of the reference value at the last visit. Changes in laboratory and vital signs after treatment will be evaluated using the descriptive statistical analysis method.

**8. Onset of hypoglycemia**

According to treatment group and visit time, the incidence of hypoglycemia in each patient will be summarized. Comparison between treatment groups will be described in the statistical analysis plan.

**9. Sub-group analysis**

If considered as appropriate, it may analyze the characteristics of each sub-group of the population (such as age, sex or current situation of treatment with oral hypoglycemic agents).

**10. Diagnostic and ranking standard for type 2 diabetes belonging to the syndrome of deficiency of both qi and yin**

The TCM symptom items were designed according to <TCM clinical diagnostic terms>, <standard for diagnosis and therapeutic effect of TCM symptoms>, the fifth edition of <TCM internal medicine>, <TCM symptom differentiation and diagnosis>, and <TCM quantization diagnosis>, on the basis of other literature and TCM commonly seen symptoms and signs of diabetes.

## XIII. Ethics requirements

Before the start of the clinical trial, the trial protocol can be implemented after reviewed and approved by the Ethics Committee and signed with suggestions for approval. During the trial, any amendment to the trial protocol can be performed only when approved by the Ethics Committee, any serious adverse events occurred in the trial should be reported to the Ethics Committee. The investigators must explain the details on the clinical trial to subjects, including:

1. The subjects are voluntary to participate in the trial, and have the right to withdraw from the trial at any time at any stage of the trial while they may not be subjected to discrimination or retaliation, and their medical treatment and interests will not be affected;

2. It must let the subjects understand that data on participation in the trial and the personal data in the trial are confidential. When necessary, the drug administrative department, the Ethics Committee or the sponsor can access the information of subjects participating in the trial according to the provisions;

3. It should inform the subjects the trial objectives, trial duration and process, inspection and operations, anticipated benefits and risks of the subjects, and inform the subjects that they may be assigned to different groups;

4. Subjects must be given sufficient time to consider whether willing to participate in the trial. For the subjects unable to express consent, it should provide their legal representative with the above description and instructions. It should use the language and words that can be understood by the subjects or their legal representative during the process of informed consent. During the trial, subjects can access the relevant information at any time;

5. If damage associated with the trial occurred, the subjects may get treatment and appropriate compensation.

The investigators will get informed consent from the subjects after full and detailed explanation of the trial. The subjects or their legal representatives will sign and date informed consent, the investigators who implement the process of informed consent also need to sign and date the informed consent form. For incapacitated subjects, if the Ethics Committee agrees in principle and the investigators consider their participation in the trial is in line with their own interests, then these patients can also enter the trial after their legal guardians agree and sign and date. In case of emergency when it is unable to get informed consent from the patients and their legal representatives, if it lacks of treatment method that has been proved as effective, while the test drug is expected to save lives, restore health, or alleviate pain, they can be considered as subjects, but it need to clearly state the method to accept these subjects in the trial protocol and relevant documents, and get prior approval from the Ethics Committee. If it founds new important information involving in the investigational drug, it must amend the informed consent in written form, which needs to obtain the subjects’ consent once again after approved by the Ethics Committee.

## IVI. Anticipated study results

1. The indicators of glycemic control in Xiaoke Pills treatment group and the glibenclamide group are similar.

2. Study of personalized therapy on the basis of molecular typing.

2.1 To identify 9-10 single nucleotide (SNPs) sites and other biomarkers (including metabolomics markers) that is guiding and promising in personalized therapy of type 2 diabetes with Xiaoke Pills in clinical practice.

2.2 To found 5-7 single nucleotide (SNPs) sites and other biomarkers (including metabolomics markers) that is associated with the special effects of Xiaoke Pills.

2.3 The efficacy of drugs To found 1-2 new important signaling molecule pathway factor that may affect the drug efficacy.

2.4 To develop a clinical protocol that can guide personalized therapy with Xiaoke Pills, improve efficacy of Xiaoke Pills and reduce side effects.

## XV. Expected progress and completion date of the clinical trial

2007.4—2007.12：

Organization of research team;

Design and preparation of detailed research protocol, Investigator’s Brochure, CRF

Implementation of research protocol and preparation of materials

Researcher training;
2008.1—2008.6

Kick-off meeting, investigator training;

Completion of enrollment and randomization

Treatment and follow-up

2009.6—2009.10

End of the trial, data analysis

**Appendix:**

**1. Diagnosis of overweight and obesity**

Chinese Diabetes Society of Chinese Medical Association suggests currently using BMI 24 and 28 recommended by China Working Group on Obesity as the diagnosis division point of overweight and obesity (Table 1). With the accumulation more precise epidemiological research data and deepening of people’s understanding of the disease, the division point will still change. For diagnosis of children and adolescents overweight/obesity, generally speaking, age-specific BMI 85 percentile or above and BMI 95 percentile or above in the BMI age-specific distribution curve of the background population are respectively defined as overweight or obese.

Table 1 Estimation of overweight/obesity in adults using body mass index

|  | BMI（kg/m2） |  | Risks of causing concomitant diseases |
| --- | --- | --- | --- |
| Classification | WHO(1998) | WHO-WPR(2000) |
| Low weight | <18.5 | <18.5 | low, but it will be increased when there are other clinical conditions |
| Normal | 18.5~24.9 | 18.5~22.9 | Common |
| Overweight | ≥25 | ≥23 |  |
| Pre-obese | 25~29.9 | 23~24.9 | increased |
| Obesity stage I | 30~34.9 | 25~29.9 | moderate |
| Obesity stage II | 35~39.9 |  | severe |
| Obesity stageIII | ≥40 |  | very severe |

Table 2 Diagnosis division point of overweight and obesity recommended by China Working Group on Obesity

| Weight | BMI（kg/m2） |
| --- | --- |
| Low weight | <18.5 |
| Normal | 18.5～23.9 |
| Overweight | 24.0～27.9 |
| Obesity | ≥28.0 |

Note: For comparability with international data, the data of BMI ≥ 25 kg/m2 and ≥ 30 kg/m2 should be calculated and included during statistics of the BMI data

2. Estimation of abdominal or central obesity (increased waist fat) using waist circumference or waist-hip ratio (WHR)

In recent years, abdominal obesity is usually diagnosed by waist circumference, but the division point used varies greatly among populations. The difference in WHR division point between populations is relatively small. Chinese Diabetes Society of Chinese Medical Association suggest currently using the waist circumference of 85 cm in males and 80 cm in females as the diagnosis division point of central obesity as recommended by China Working Group on Obesity (Table 3).

Table 3 Relationship between BMI and waist circumference with relative risk of obesity-related diseases of China *

| BMI（kg/m2） | Waist circumference（cm） |
| --- | --- |
|  | Males≤85 >85  Females≥80 >80 |
| <18.5  18.5～23.9 increase  24.0～27.9 Increase High  ≥28.0 high very high | |

* Relative risk refers to the risk of type 2 diabetes, hypertension and cardiovascular disease as compared with those with normal BMI and waist circumference,

**Xiaoke Pills Package Insert**

**Drug name**

Common name: Xiaoke Pills

Pinyin: Xiaoke Wan

**INGREDIENTS:**

Radix Puerariae, Radix Rehmanniae, Radix Astragali, Radix Trichosanthis, Stylus Zeae Maydis, Fructus Schisandrae Sphenantherae, Rhizoma Dioscoreae and Glibenclamide.

DESCRIPTION:

Black coated concentrated pills; sweet, sour and slightly astringent in taste.

**ACTIONS AND INDICATIONS:**

Nourish kidney-*yin*, replenish *qi*, promote generation of fluid. Indicated for diabetes due to deficiency of *qi* and *yin* (type 2 diabetes) manifested as polydipsia, polyphagia and polyuria, emaciation , fatigue, shortness of breath, indolent about speaking.

SPECIFICATION:

Per 10 pills weight 2.5g (contains glibenclamide 2.5mg).

**USAGE AND DOSAGE:**

For oral use before meals. 5-10 pills twice or thrice daily or by medical order.

**ADVERSE REACTION:**

Not known yet.

**CONTRAINDICATIONS:**

1. It is contraindicated for lactating women, so as to prevent infant from hypoglycemia.
2. Contraindicated for pregnant women, patients with insulin-dependent diabetes, patients with non-insulin dependent diabetes accompanied by ketoacidosis, coma, severe burns, infections, severe trauma and patients undergoing major surgery. Contraindicated for patients with hepatic and renal insufficiency, allergy of sulfonamides and leucopenia.
3. It should be used with caution for patients with weak constitution, high fever, nausea and vomiting, the elderly, patients with hypoadrenocorticism or impaired anterior pituitary function.

**Precautions:**

The product is a compound preparation of Chinese herbs combined with western medicine (glibenclamide). In view of shortage of clinical data, it is difficult to prove that this compound preparation can reduce or eliminate the adverse reaction of glibenclamide. The following items list the correlative information to physicians and patients.

1. This product contains glibenclamide. Glibenclamide is an oral hypoglycemic agent belonging to the class of sulfonylureas. In monotherapy, the daily dose of glibenclamide starts from 2.5 mg, while 1.25 mg for mild patients, once before breakfast or each once before breakfast and lunch, thereafter, the dose is adjusted according to the efficacy every other week, the common dosage is 5-10 mg/day, and the maximal dose is 15 mg/day.
2. The main reactions of glibenclamide monotherapy include: (1) hypoglycemia. (2) gastrointestinal reactions, such as nausea, vomiting, diarrhea, and metallic taste in the mouth. (3) allergic reactions such as rash, occasionally exfoliative dermatitis. (4) hematologic abnormalities such as leukopenia, agranulocytosis, anemia, thrombocytopenia. (5) liver damage, such as jaundice, liver damage.
3. Drug interactions with glyburide: increase the incidence of hypoglycemia: (1) probenecid, allopurinol. (2) alcohol, cimetidine, ranitidine, chloramphenicol, miconazole, coumarin anticoagulants, lipid-lowering drugs fibrates. (3) salicylic acid, guanethidine, monoamine oxidase inhibitor, quinidine. (4) insulin, metformin, acarbose, insulin sensitizer. (5) β-adrenal receptor blocking agent can interfere the increase of blood sugar during hypoglycemia and block the zymolysis of hepatic glycogen and cover the hypoglycemic symptoms simultaneously. Increase the incidence of hyperglycemia: (1) glucocorticoids, estrogen, thiazide diuretics, phenytoin, rifampin. (2) β-adrenergic receptor blockers can antagonize the effect of sulfonylureas on insulin secretion, so it also can cause hyperglycemia.
4. During the medication, regularly test the blood sugar, urine glucose, urine acetone bodies, urine protein, hepatic and renal function, hemogram and perform ophthalmic examination.

**PHARMACOKINETICS:**

The test of pharmacokinetics showed that the blood concentration of glibenclamide increased after 30 minutes of administration and after 2-3 hours attained to highest level.

**STORAGE:**

Hermetically sealed.

**Packaging:**

Packaged in plastic bottles, 30 grams (120 pills) per bottle, 1 bottle per box.

**Shelf life:**

36 months.

**Executive standard**:

State Food and Drug Administration Standard (Trial) YBZ11322006

**production batch number:**

K03300

**Approval No.:**

SFDA (approved) Z44020045

**Manufacturer:**

Company Name: Guangzhou Zhongyi Pharmaceutical Co., Ltd.,

Address: No. 50 of Conggui New Street, Conggui Road, Guangzhou City,

Zip Code: 510140

**Glibenclamide tablets package insert**

**Drug name**

Common name：Glyburide tablets

Pinyin：GELIEBENNIAO PIAN

English name：GLIBENCLAMIDE TABLETS

**INGREDIENTS:**

Glibenclamide

**Drug category**

Insulin and other drugs affecting blood glucose

**Discription**

This product is a white tablet.

**Pharmacology and toxicology**

This product is a hypoglycemic agent. 1. To stimulate the pancreatic islet β cells to secret insulin, under a prerequisite that the pancreatic islet β cells still have certain function of synthesis and secretion of insulin; 2. To inhibit hepatic glycogen breakdown and gluconeogenesis role by increasing insulin levels in portal vein or direct effects on the liver, reducing liver glucose production and output; 3. may also increase the sensitivity of external pancreatic tissue to insulin and glucose utilization (mainly through the post-receptor effect), so the overall effect is to reduce fasting and postprandial blood glucose.

**Pharmacokinetics**

Rapid oral absorption, high protein binding rate of 95%, plasma concentration reaching the peak 2-5 hours after oral administration, sustained for 24 hours. Half-life is 10 hours. Metabolized in the liver. About 50% excreted by the liver and kidneys each.

**Indications**

It is indicated for mild to moderate type 2 diabetes with unsatisfactory efficacy by diet control alone, while patients’ pancreatic β cells have certain function to secrete insulin, without serious complications.

**Usage and dosage**

Take orally at an initial dose of 2.5 mg, once before breakfast or each once before breakfast and lunch; for mild patients, take a dose at 1.25 mg, three times a day before meals, and increase the dose to 2.5 mg after 7 days. The common daily dosage is 5-10 mg, and the maximum daily dosage does not exceed 15mg.

**Adverse reactions**

1. It may cause diarrhea, nausea, vomiting, headache, stomach pain or discomfort; 2. skin rash is less commonly seen; 3. severe jaundice, liver function damage, bone marrow suppression, neutropenia (manifested as a sore throat , fever, infection), and thrombocytopenia (manifested as bleeding, purpura) are rarely seen.

**Contraindications**

It is contraindicated in the following conditions: 1. typeⅠdiabetes; 2. type 2 diabetes associated with stress conditions such as ketoacidosis, coma, severe burns, infection, trauma and major surgery; 3. patients with liver and kidney dysfunction; 4. Patients allergic to sulfonamide; 5. patients with neutropenia.

**Announcements**

It should be used with caution in the following conditions: weak constituation, high fever, nausea and vomiting, hyperthyroidism, elderly. 2. It should regularly measure the blood sugar, urine sugar, urine ketone, urine protein, liver and kidney function, and conduct eye examination during medication.

**Medication on pregnant and lactating women**

1. Animal experiments and clinical observation showed sulfonylurea hypoglycemic agents can cause stillbirths and fetal anomaly, so pregnant women should not take it.

2. The kind of drugs can be excreted through the breast milk, thus lactating mothers should not take it to prevent infant hypoglycemia.

**Medication for elderly patients**

The elderly or patients or with renal dysfunction have reduced function on metabolism and excretion of this kind of drugs, and this product has relative stronger hypoglycemic effects, so they should not use this product, but can use other sulfonylurea hypoglycemic agents of shorter duration of action.

**Drug Interactions**

1. When taken with alcohol, it can cause abdominal cramps, nausea, vomiting, headache, facial flushing and hypoglycemia. 2. When in combination with β-receptor blockers, it can increase the risk of hypoglycemia, and can cover the symptoms of hypoglycemia, such as increased pulse rate and blood pressure; a small dose of selective β- receptor blockers, such as atenolol and metoprolol, are less likely to cause such reactions. 3. This product in combination with Chloramphenicol, guanethidine, insulin, monoamine oxidase inhibitors, phenylbutazone, hydroxyl phenylbutazone, probenecid, salicylates, and sulfonamides may enhance the hypoglycemic effects.4. Adrenocorticotropic hormone, epinephrine, phenytoin, thiazide diuretics, and thyroxine can increase blood glucose levels, When used in combination with this kind of drugs, it may need to increase the dosage. 5. When coumarin anticoagulants is used in combination with this product, the initial plasma concentration of both drugs will be increased first, but later the plasma concentration of both will be reduced, so it need to adjust the dosage of both.

**Storage**

Stored airtight for preservation

**Packaging**

2.5milligrams per tablet, 100 tablets per bottle

**Shelf life:**

3 years.

**PRODUCTION BATCH NO.:**

070708

**Approval No.:**

SFDA (approved) H12020790

**Manufacturer:**

Company Name: Tianjin Pacific Pharmaceutical Co., Ltd

Address: No 17 of Jiefang South Street, Outer Ring Road, Tianjin City

Zip Code: 300381

**Metformin hydrochloride tablets package insert**

**Drug name**

Common name：Metformin hydrochloride tablets

Pinyin：YANSUAN ERJIASHUANGGUA PIAN

English name：METFORMIN HYDROCHLORIDE TABLETS

**Major ingredient**

1,1-dimethyl biguanide hydrochloride

Structural formula:


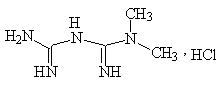


**Drug category**

Insulin and other drugs affecting blood glucose

**Description**

This product is a white tablet.

**Pharmacology and toxicology**

This product is hypoglycemic agent. It can decrease the fasting and postprandial blood glucose for patients with type 2 diabetes, while HbAlc can be decreased by 1% to 2%. The hypoglycemic mechanism of this product may be: 1. Increase the peripheral tissues’ sensitivity to insulin, increased insulin-mediated glucose use. 2. Increase glucose utilization by non-insulin-dependent tissues such as the brain, blood cells, kidney medulla, intestine, skin, and so on. 3. Inhibit gluconeogenesis effect of liver glycogen, decrease hepatic glucose output. 4. Inhibit glucose intake by the intestinal wall cells. 5. Inhibit biosynthesis and storage of cholesterol, lower blood triglycerides and total cholesterol levels. Different from the effects of insulin, this product has no effect to promote fat synthesis, and has no significant hypoglycemic effects on normal subjects, and generally does not cause hypoglycemia when used alone in type 2 diabetes.

**Pharmacokinetics**

Metformin is mainly absorbed by small intestine, the absorption half-life is of 0.9~2.6 hours, with bioavailability of 50%~60%. 2 hours after oral administration of metformin 0.5g, its plasma concentration reaches peak, nearly 2 μg/ml. A high level of metformin is concentrated within the gastrointestinal tract wall, which is 10~100 times the plasma concentration. The content in kidney, liver and saliva is approximately 2 times the plasma concentration. Metformin has a stable structure, it does not bind to plasma proteins, it is excreted in prototype through urine, and cleared rapidly, with plasma half-life of 1.7~4.5 hours, and cleared by 90% within 12 hours. As part of this product can be secreted by the renal tubules, its renal clearance rate is greater than the glomerular filtration rate, as the product is mainly excreted in prototype by the kidneys, so if this product is used when the renal function is dereased, it can largely accumulate in the body, causing hyperlactatemia or lactic acidosis.

**Indications**

When used in type 2 diabetes with unsatisfactory glycemic control by dietary management alone, particularly associated with obesity and hyperinsulinemia, this drug can not only lower blood sugar, but also may lose weight and relieve hyperinsulinemia. It may be effective in some patients showing poor efficacy after treated with sulfonylureas. If it is used in combination with sulfonylureas, intestinal glucosidase inhibitor or thiazolidinedione diketone antidiabetic agents, the effects are better than monotherapy. It can also be used in patients receiving insulin therapy to reduce the dosage of insulin.

**Usage and dosage**

For oral administration. Adults start at an dose of 0.25g, 2 or 3 times a day; afterward gradually increase the dose according to the efficacy, generally 1~1.5g a day, and the maximal daily dose should not exceed 2g. Oral administration at meals or immediate oral administration at meals can reduce gastrointestinal reactions.

**Adverse reactions**

1. Common: nausea, vomiting, diarrhea, metallic taste in mouth. 2. Occasional: weakness, fatigue, dizziness, skin rash. 3. Although the incidence of lactic acidosis is low, but it should be noted. Clinical manifestations include vomiting, abdominal pain, hyperventilation, consciousness disorder, increased blood lactate concentration which can not be explained using uremia, ketoacidosis, or salicylic acid poisoning. 4. It can reduce the intestinal absorption of vitamin B12, thus reducing hemoglobin, resulting in megaloblastic anemia, and it also can cause malabsorption.

**Contraindications**

It is contraindicated in the following situations: 1. Type 2 diabetes associated with ketoacidosis, liver and renal dysfunction (serum creatinine more than 1.5mg/dl), pulmonary insufficiency, heart failure, acute myocardial infarction, severe infection and trauma, major surgery and clinical hypotension and hypoxia. 2. Diabetic patients with severe chronic complications (such as diabetic nephropathy, diabetic retinopathy). 3. Before intravenous pyelography or angiography. 4. Alcoholics. 5. with serious heart and lung diseases 6. Patients lack of Vitamin B12, folic acid and iron. 7. Patients with poor general condition (such as malnutrition, dehydration).

**Announcements**

1. This product should not be used alone (can be combined with insulin) in type Ⅰdiabetes. 2. During medication, it should regularly check blood glucose, urine glucose and urine ketone bodies, and regularly measure serum creatinine and blood lactate concentration.

3. When it is used in combination with insulin, it should prevent the occurrence of hypoglycemia reactions.

**Medication on pregnant and lactating women**

It should not be used in pregnant and lactating women drug.

**Medication on the elderly**

It should be used with cautious in elderly patients (> 65 years), as their renal function is decreased, so the dosage should be reduced.

**Drug Interactions**

1. This product in combination with insulin will enhance the hypoglycemic effect, so it should adjust the dose. 2. It can be enhance the anticoagulant effects of anticoagulant drugs (such as warfarin, etc.), leading to bleeding tendency. 3. Cimetidine can increase the bioavailability of the product and reduce the renal clearance, therefore, it should reduce the dose of this product when used in combination with cimetidine.

**Storage**

Storage sealed.

**Shelf life:**

3 years.

**PRODUCTION BATCH NO.**

070908

**Approval No.**

SFDA (approved) H11020976

**Manufacturer:**

Company Name: Beijing Union Pharmaceutical Co., Ltd

Address: Xingye North Road, Huangcun Town, Daxing District, Beijing

Zip Code: 102600

**References**

1. SFDA. Regulation on Drug Registration, May, 2005

2. Guiding Principles for Clinical Study of New Western Medicine. Beijing: P.R.C. Ministry of Health Bureau of Pharmaceutical Affairs; 1993

3 Zheng Xiaoyu. Guiding Principles for Clinical Study of New Chinese Medicine (on trial. Beijing: Chinese Medical Science and Technology Press; 2002

4. SFDA. Good Clinical Practice. September 2003

5. SFDA. Provisions on Clinical Research of Drugs. July 2000

6. SFDA. Technical Requirements for New Chinese Medicine Research. November 1999

7. P.R.C. TCM Industry Standards—Diagnosis and Efficacy Standards for TCM Syndromes. State Administration of Traditional Chinese Medicine ZY/001.6 1995:126

8 Lai Shilong. Chinese Medicine Clinical Trials. Guangdong: Guangdong People's Publishing House, 2001; Chen Haozhu. Practical Internal Medicine. Beijing: People's Medical Publishing House; 2004

9. Xiaoke Pills Package Insert

10.Glibenclamide Tablets Package Insert

11. Metformin Hydrochloride Tablets Package Insert
