# Supplementary material for: Efficacy and Safety of Traditional Chinese Medicine for Diabetes: A Double-Blind, Randomised, Controlled Trial
Source: PLoS One. 2013 Feb 27;8(2):e56703. doi: 10.1371/journal.pone.0056703 (PMC3584095; doi:10.1371/journal.pone.0056703)
Supplement: Appendix S1 — List of Investigators. (DOC) [file pone.0056703.s006.doc]

**List of Investigators**

Investigator:

The First Hospital of Hebei Medical University, Shijiazhuang, China: Huimin Zhou (principal investigator), Yuqing Guo.

The Third Affiliated Hospital of Peking University of Traditional Chinese and Western Medicine, Beijing, China: Lili Zhang (principal investigator), Shan Li, Xiaopeng Zhao, Jianqiang Ma.

Sichuan University West China Hospital, Chengdu, China: HaomingTian (principal investigator), Zhenmei An, Shaorui Shi, Mei Zhang, Lin Zhang.

The First Affiliated Hospital of Chongqing Medical University, Chongqing, China: Qifu Li (principal investigator), Zhihong Wang, Lilin Gong, Zhiping Liu, Xiaojuan Tan.

The Central Hospital of China Aerospace Corporation, Beijing, China: Yizhong Wang (principal investigator), Xiaolin Jia, Yufang Li, Song Dong, Danping Meng.

Peking University People’s Hospital, Beijing, China: LinongJi (principal investigator), Xuyao Han, Yingli Chen, Xianghai Zhou, Lingli Zhou, Jing Chen, Hong Wang, Qian Ren, Wei Liu.

China Meitan General Hospital, Beijing, China: Hongmei Li (principal investigator), Yang Han, Jun Zhang, Yan Sun, Kailiang Wang.

The First Affiliated Hospital of Guangzhou University of Traditional Chinese Medicine, Guangzhou, China: Min Liu (principal investigator), Zhizhang Zhu, Fangfang Wang, Chuangpeng Shen, Yan Zhang.

Shanghai University of Traditional Chinese Medicine, Yueyang Hospital of Integrated Traditional Chinese and Western Medicine, Shanghai, China: Hongjie Yang (principal investigator), Chaohua Fan, Min Zheng, Zhang Dan, Junhao Li.

China Academy of Chinese Medicial Sciences Guang’an men Hospital, Beijing, China: Xiaolin Tong (principal investigator), Qing Ni.

Beijing University of Traditional Chinese Medicine Dongfang Hospital, Beijing, China: Yanbin Gao (principal investigator), Xuezheng Shang, Tong Sheng.

Zhongshan University Sun Yai-sen Memorial Hospital, Guangzhou, China: Yan Li (principal investigator), Chuan Yang, Fangping Li, Huisheng Xiao, Muchao Wu.

General Hospital of PLA Second Artillery, Beijing, China: Quanmin Li (principal investigator), Xiran Wang, Ruiqin Du, Qiulan Zhang, Yanqiu Zhu.

Peking University First Hospital, Beijing, China: Xiaohui Guo (principal investigator), Peihong Jia, Xiaojing Zhou, Yan Hui.

The Second Affiliated Hospital of Chongqing Medical University, Chongqing, China: Gangyi Yang (principal investigator), Ling Li, Wenwen Chen.

Nanjing Hospital of Traditional Chinese Medicine, Nanjing, China: Zhongai Zhang (principal investigator).

The Second Xiangya Hospital of Central South University, Changsha, China: Zhiguang Zhou (principal investigator), Weili Tang, Shiping Liu, Hui Mo, LifeiFeng.

Shanghai Jiaotong University Ruijin Hospital, Shanghai, China: GuangNing (principal investigator), Weiqing Wang, Jie Hong, Weiqiong Gu, Yifei Zhang.
